# Removal of TREX1 activity enhances CRISPR–Cas9-mediated homologous recombination

**DOI:** 10.1038/s41587-024-02356-3

**Published:** 2024-08-12

**Authors:** Mehmet E. Karasu, Eléonore Toufektchan, Yanyang Chen, Alessandra Albertelli, Grégoire Cullot, John Maciejowski, Jacob E. Corn

**Affiliations:** 1 Department of Biology, Institute of Molecular Health Sciences, Swiss Federal Institute of Technology (ETH) Zurich, Zurich, Switzerland; 2 Molecular Biology Program, Sloan Kettering Institute, Memorial Sloan Kettering Cancer Center, New York, NY USA

**Keywords:** Homologous recombination, Genetic engineering, Targeted gene repair

## Abstract

CRISPR–Cas9-mediated homology-directed repair (HDR) can introduce desired mutations at targeted genomic sites, but achieving high efficiencies is a major hurdle in many cell types, including cells deficient in DNA repair activity. In this study, we used genome-wide screening in Fanconi anemia patient lymphoblastic cell lines to uncover suppressors of CRISPR–Cas9-mediated HDR. We found that a single exonuclease, *TREX1*, reduces HDR efficiency when the repair template is a single-stranded or linearized double-stranded DNA. *TREX1* expression serves as a biomarker for CRISPR–Cas9-mediated HDR in that the high *TREX1* expression present in many different cell types (such as U2OS, Jurkat, MDA-MB-231 and primary T cells as well as hematopoietic stem and progenitor cells) predicts poor HDR. Here we demonstrate rescue of HDR efficiency (ranging from two-fold to eight-fold improvement) either by *TREX1* knockout or by the use of single-stranded DNA templates chemically protected from TREX1 activity. Our data explain why some cell types are easier to edit than others and indicate routes for increasing CRISPR–Cas9-mediated HDR in *TREX1*-expressing contexts.

## Abstract (web summary)

Homologous recombination in CRISPR–Cas9 genome editing is increased by blocking an exonuclease activity.

## Main

Repair of CRISPR–Cas9-induced double-strand breaks (DSBs) occurs by one of two main mechanisms: non-homologous end joining (NHEJ) or homology-directed repair (HDR)^1–3^. The HDR pathway is used to introduce sequences of up to 2–3 kilobases (kb) into the genome that are supplied on an exogenous DNA template. In most human cells, the efficiency of HDR is very low relative to NHEJ^4,5^. Cellular context plays an important role in HDR efficiency. HDR is cell cycle limited and most active in S/G2 (refs. ^6–8^). Differential expression, cellular background and/or mutational burden can also affect efficiency. CRISPR–Cas9-mediated targeting of the same locus with identical reagents in various cell types can result in variable efficiency, ranging from 30% of alleles to complete inactivity^9^. The efficiency of prime editing is determined by the mismatch repair status of the targeted cells, yet a similar biomarker for CRISPR–Cas9-mediated HDR efficiency is currently unknown^10,11^.

Patient cells carrying mutations in DNA repair genes can also compromise CRISPR–Cas9-mediated genome editing, complicating efforts to correct the targeted disorder. For example, loss of function in one of 22 genes involved in Fanconi anemia (FA) can largely prevent HDR^9,12,13^. FA is a rare genetic disorder characterized by bone marrow failure and predisposition to malignancies later in life^14^. Attempts to correct FA patient mutations by CRISPR–Cas9-induced HDR have revealed poor efficiencies, limiting potentially curative genome editing approaches to those that circumvent HDR but are not applicable to all FA alleles^15,16^.

In the present study, we performed genome-wide screening in lymphoblastic cell lines (LCLs) derived from patients with FA to uncover factors that restrict CRISPR–Cas9-mediated HDR. We found that TREX1, a widely expressed endoplasmic reticulum (ER)-associated nuclease involved in innate immunity, plays a dominant role in reducing CRISPR–Cas9-induced HDR in human cells. Knockout of *TREX1* rescues HDR in FA patient-derived cells and in commonly used cell models with naturally low HDR efficiency. Chemical protection of DNA donor templates in a manner designed to prevent TREX1 activity rescues HDR in these TREX1-expressing cells at multiple loci. Our work highlights the importance of cellular factors in regulating genome editing outcomes, provides a rational explanation of the seemingly stochastic efficiency of CRISPR–Cas9-mediated HDR in various cell backgrounds and offers a potential path to high levels of genome editing in the myriad cell models and primary cell lines limited by TREX1 expression.

## Results

### Removal of TREX1 reactivates HDR in FA patient cells

**Fig. 1:**
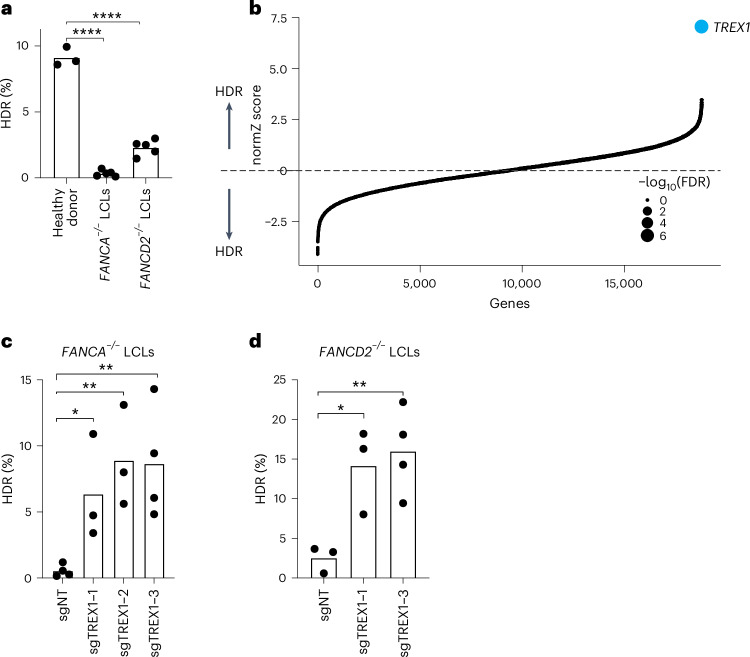
Identification of TREX1 as a restrictive factor of CRISPR–Cas9-mediated HDR. **a**, FA patient-derived LCLs are compromised in a BFP-to-GFP reporter assay for CRISPR–Cas9-mediated HDR. The efficiency of HDR was measured by flow cytometry 5 d after Cas9 targeting (*n* = 5 for FA LCLs and *n* = 3 for healthy donor, biologically independent experiments, *P* = 1.94 × 10^−7^ and 6.0 × 10^−6^). **b**, Genome-wide CRISPRi/CRISPRn screening identified *TREX1* as the sole gene whose knockdown strongly rescues HDR in *FANCA*^−/−^ LCLs. Gene-level effects and statistics were calculated using drugZ and ranked by the normZ score. The size of each point reflects the FDR (Supplementary Table 2). **c**,**d**, CRISPRi knockdown of *TREX1* significantly increases CRISPR–Cas9-mediated HDR in *FANCA* (**c**) and *FANCD2* (**d**) deficient LCLs. Both cell backgrounds were stably transduced with up to three different sgRNAs targeting *TREX1*, and a BFP-to-GFP assay was used to measure HDR efficiency. In Fig. 1a,c,d, each dot represents an individual biological replicate, and bars represent the mean (*n* = 4 for sgNT in **c** and sgTREX1-3 in **d**, *n* = 3 for the rest of the samples in **c** and **d**, biologically independent experiments, *P* = 0.031, 0.0065 and 0.0088 for **c** and *P* = 0.0234, 0.0096 for **d**, respectively). All *P* values were calculated using an unpaired and two-sided *t*-test, *P < 0.05, **P < 0.01, ***P < 0.001. NT, non-targeting. Source data are provided as a Source Data file. Source data

We first analyzed CRISPR–Cas9-mediated HDR efficiencies of LCLs from *FANCA*^*–/–*^ and *FANCD2*^*–/–*^ patient backgrounds with a previously published BFP-to-GFP reporter^9,17,18^. This BFP sequence can be targeted by a Cas9 ribonucleoprotein (RNP) and an appropriate single-stranded oligodeoxynucleotide (hereafter ssODN) HDR template to convert BFP-His151 to GFP-Tyr151 (ref. ^18^). After optimizing electroporation conditions in healthy donor LCLs (Supplementary Fig. 1, top), we verified that HDR efficiencies in FA LCLs were extremely low compared to wild-type LCLs, especially in the *FANCA*^*–/–*^ background (for example, 0.3 ± 0.2% *FANCA*^*–/–*^ versus 2.3 ± 0.6% *FANCD2*^*–/–*^ versus 9.1 ± 0.7% wild-type) (Fig. 1a). We, therefore, searched for factors whose removal could rescue HDR in *FANCA*^*–/–*^ cells.

We used pooled genetic screening to uncover factors regulating CRISPR–Cas9-mediated HDR^17,19,20^ (Supplementary Fig. 1, bottom). We employed a previously published BFP-to-GFP HDR reporter and paired CRISPR inhibition (CRISPRi) and CRISPR RNP system to knock down one gene per cell while simultaneously inducing a DSB and a paired ssODN repair template at the reporter locus^9,21^. Fluorescence-activated cell sorting (FACS) for conversion of BFP to GFP and sequencing of the recovered single guide RNAs (sgRNAs) was used to reveal factors that modulate HDR. Technical concerns limited our prior work in using this screening system to a set of 2,000 core genes involved in DNA metabolism^9^. In the present study, we scaled up the previous screening strategy 10-fold to a genome-wide level in a much more difficult patient-derived cell background (Methods).

*FANCA*^*–/–*^ LCLs were first lentivirally engineered to stably express KRAB–dCAS9–mCherry (CRISPRi). We recloned all guide RNAs (gRNAs) in the CRISPRiv2 library^22^ to a modified reporter lentiviral vector with BFP placed downstream of the sgRNA cassette. During screening, electroporation of Cas9 RNP and ssODN into *FANCA*^*–/–*^ CRISPRi LCLs yielded a GFP^+^ fraction of approximately 0.5% (Supplementary Fig. 2). We used a dual-thresholding and pure-sort strategy to rapidly yet accurately isolate the rare cells that were performing high levels of HDR (Methods). We amplified sgRNAs by polymerase chain reaction (PCR) from the unsorted and GFP^+^ populations after biological duplicate viral transductions, sequenced the population of sgRNAs using next-generation sequencing (NGS), quantified guide abundances using MaGECK^23^ and calculated gene-level enrichment scores and significance using drugZ^24^. We found that a single gene called *TREX1* was highly enriched in the GFP^+^ HDR population (false discovery rate (FDR) < 1 × 10^−7^) (Fig. 1b and Supplementary Table 1).

### TREX1 destabilizes unprotected HDR templates

TREX1 is a 3′-to-5′ exonuclease that is anchored to the outer membrane of the ER and is involved in suppressing chronic activation of cyclic GMP–AMP synthase (cGAS) during the innate immune response to cytosolic DNA^25–27^. TREX1 is active on single-stranded and double-stranded DNA molecules and has a 1,000-fold lower activity toward RNA and RNA–DNA hybrids^25,28^. Mutations associated with TREX1 lead to autoimmune disorders, such as Aicardi–Goutières syndrome^29–31^. Notably, neither TREX2 (46% identical to TREX1 in the catalytic domain) nor the hundreds of other nucleases present in human cells were screening hits.

To validate the primary screen result, we individually cloned multiple gRNAs against *TREX1* and performed individual CRISPRi in both *FANCA*^*–/–*^ and *FANCD2*^*–/–*^ LCLs. Quantification of baseline expression of *TREX1* by qRT–PCR revealed very high expression in both FA backgrounds relative to healthy donor cells (Supplementary Fig. 3, top). CRISPRi knockdown was effective with multiple *TREX1*-targeting gRNAs measured by qRT–PCR and western blotting (Supplementary Fig. 3, bottom). Individual BFP-to-GFP reporter assays showed that *TREX1* knockdown restored HDR activity in both *FANCA*^*–/–*^ and *FANCD2*^*–/–*^ patient backgrounds (Fig. 1c and Supplementary Fig. 4).

**Fig. 2:**
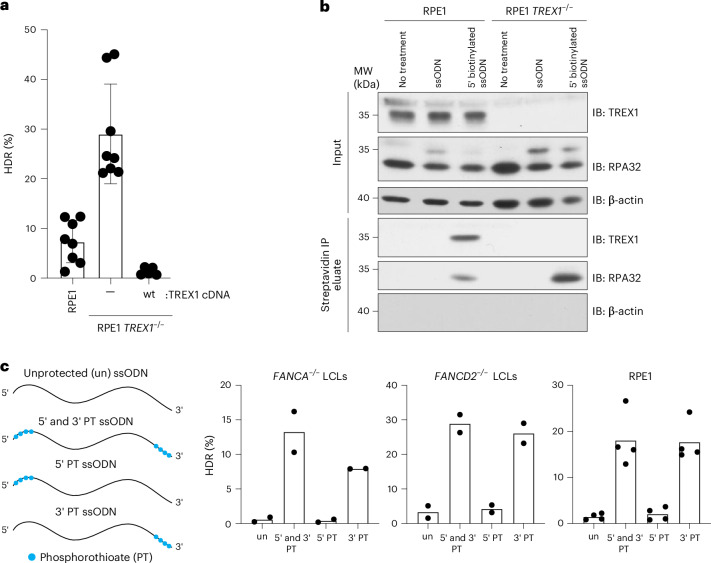
TREX1 interacts with ssODN HDR templates and is inhibited by phosphorothioate protection. **a**, RPE-1 hTERT *TREX1*^*–/–*^cells perform high levels of HDR, and *TREX1* cDNA complementation abrogates HDR as measured by the BFP-to-GFP assay. Dots (*n* = 8 for RPE1 wild-type and RPE-1 *TREX1*^*–/–*^, *n* = 5 for RPE-1 hTERT *TREX1*^*–/–*^ + wild-type TREX1 cDNA) represent individual biological replicate measurements; bars represent the mean values; and error bars represent the standard deviation. **b**,TREX1 co-immunoprecipitates with 5′-biotin-labeled ssODN template. HDR donors were delivered by electroporation to RPE-1 hTERT wild-type and RPE-1 hTERT *TREX1*^*–/–*^ cells. After 20 min, cells were collected, and lysates were prepared for immunoprecipitation with streptavidin beads. Blots were probed with anti-TREX1, anti-RPA32 and anti-β actin (*n* = 1). **c**, Incorporation of four phosphorothioate (PT) bonds on the 5′ and 3′ or only 3′ ends of an ssODN rescues HDR efficiency in *FANCA*^*–/–*^ LCLs, *FANCD2*^*–/–*^ LCLs and RPE1 cells. 5′ end protection behaves as unprotected (un) ssODN. HDR was measured using the BFP-to-GFP assay. Each dot represents an individual biological replicate (*n* = 2 for *FANCA*^*–/–*^ LCLs and *FANCD2*^*–/–*^ LCLs and *n* = 4 for RPE-1 hTERT cells), and bars represent the mean. Source data are provided as a Source Data file. IB, immunoblotting; IP, immunoprecipitation; wt, wild-type. Source data

We further tested the effect of TREX1 by using CRISPR–Cas9 to create an isogenic *TREX1* knockout in RPE-1 hTERT cells, which have otherwise functional DNA repair^32^. We then ectopically expressed wild-type TREX1 in the knockout clone^32^ (Supplementary Fig. 5). A BFP-to-GFP assay showed that wild-type RPE1 cells perform moderate levels of CRISPR–Cas9-induced HDR; *TREX1* knockout strongly increased HDR; and overexpression of wild-type *TREX1* in the knockout background almost completely abrogated HDR (Fig. 2a).

Given TREX1’s known activity as an exonuclease, it could either be altering repair profiles at the break^33^ or interacting with and degrading the DNA repair template used during CRISPR–Cas9-mediated HDR. We performed editing experiments in wild-type and TREX1-depleted HeLa cells and analyzed the indel changes around the potential DSB site at two independent loci. However, we did not find substantial changes in indel formation, especially large deletions (Supplementary Fig. 6).

We, therefore, asked whether TREX1 physically interacts with an ssODN delivered to cells. We electroporated *TREX1* wild-type and *TREX1*^*–/–*^ RPE-1 hTERT cells with a 5′ biotinylated ssODN template and performed immunoprecipitation using streptavidin beads 2 h after nucleofection. As a positive control, we blotted for RPA32, which interacts strongly with ssODNs^34^. RPA32 was readily measurable in the immunoprecipitated samples from both *TREX1* wild-type and *TREX1*^*–/–*^ cells. *TREX1* was also strongly associated with the biotinylated ssODN in the *TREX1* wild-type sample, demonstrating that TREX1 interacts with electroporated ssDNA templates and suggesting that TREX1 may directly reduce their availability for HDR (Fig. 2b).

TREX1 is an ER-associated enzyme, with its nuclease domain facing to cytosol^25,32,35^. Hence, TREX1 probably degrades cytoplasmic DNA templates before their diffusion into the nucleus. However, some studies suggested that TREX1 may be actively involved in DNA repair and could shuttle to the nucleus after DNA damage^25,36^. Our data using protected ssODNs indicate that TREX1’s main role in restricting CRISPR–Cas9-mediated HDR is related to template availability. Recent studies also found that TREX1 associates with transfected DNA in the cytosol^37^. To further investigate whether TREX1 localization was affected during CRISPR–Cas9 editing, we analyzed the localization of TREX1 at 24 h after multiple forms of CRISPR–Cas9 editing. We found that TREX1 remained outside the nucleus in all conditions (Supplementary Fig. 7). A Cas9-mediated DSB is, therefore, insufficient to induce substantial changes in TREX1 localization. Future live-cell studies could address the exact subcellular location where TREX1 degrades the majority of DNA templates during genome editing.

If TREX1 is indeed a prominent exonuclease affecting the stability of ssODN templates, a 3′ protected ssODN should block its 3′-to-5′ hydrolytic activity^38^. We, therefore, measured HDR efficiency using protected ssODNs that have phosphorothioate bonds between five nucleotides at the 5′ and/or 3′ ends (Fig. 2c). An unprotected ssODN yielded moderate HDR in wild-type RPE-1 hTERT cells and very low HDR in *FANCA*^*–/–*^ and *FANCD2*^*–/–*^ LCLs. Protection of the ssODN at both the 5′ and 3′ ends markedly increased HDR in all cell types without otherwise affecting molecular indel profiles (Supplementary Fig. 8). Consistent with the polarity of TREX1 activity, individual 3′ end protection was sufficient to increase HDR, whereas 5′ end protection performed similarly to the unmodified ssODN.

### TREX1 restricts HDR efficacy in multiple cell backgrounds and is circumvented by template protection

We wondered if TREX1 could explain the widely variable CRISPR–Cas9-mediated HDR efficacy observed in different human cell types. *TREX1* expression is reported to differ widely between cell lines, which we confirmed by qRT–PCR (Supplementary Figs. 9 and 10). Cell lines commonly used for genome editing with anecdotally high levels of HDR, including K562 and HEK293, have low levels of *TREX1*. In contrast, cells where HDR is much more difficult, such as U2OS and Jurkat, express high levels of *TREX1*. We explicitly tested this observation in several ways.

**Fig. 3:**
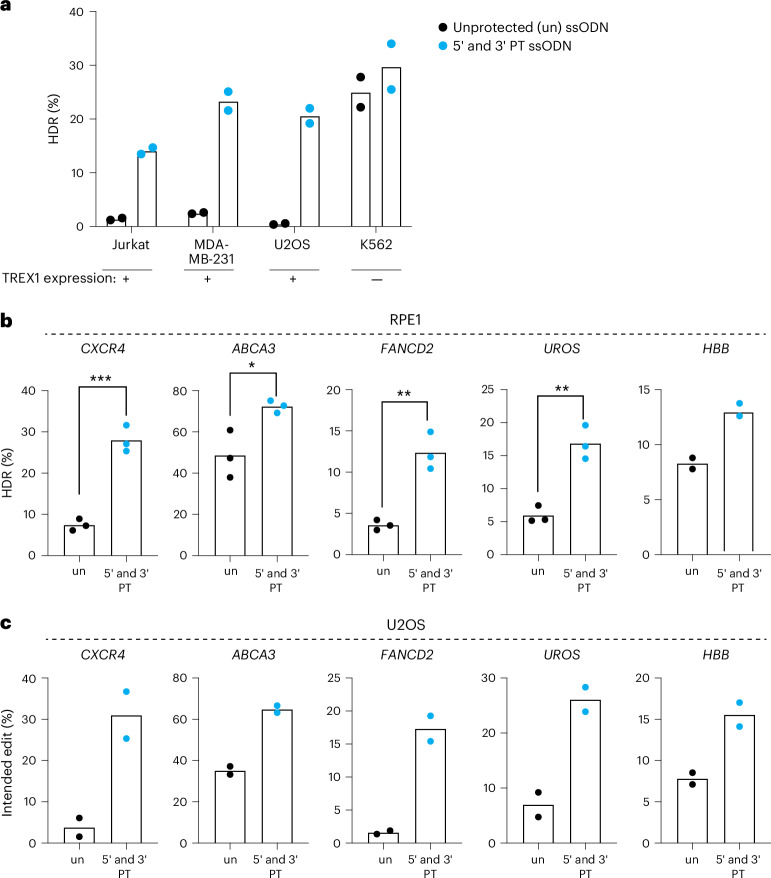
Protected ssODN templates increase HDR in *TREX1*-expressing cell contexts at multiple loci. **a**, Cell types expressing *TREX1* exhibit compromised HDR, but this is rescued by using phosphorothioate-protected ssODNs. A cell type with normally low levels of *TREX1* is already efficient at CRISPR–Cas9 HDR, and this is not further improved by a protected ssODN. HDR was measured by the BFP-to-GFP assay in Jurkat, MDA-MB-231, U2OS and K562 cell lines. Black dots represent measurements with unprotected (un) ssODN template, and blue dots represent measurements with 5′ and 3′ protected (PT) ssODN templates. Each dot represents individual biological replicate measurements (*n* = 2, biologically independent experiments). **b**,**c**, CRISPR–Cas9-mediated HDR efficiency is rescued at multiple endogenous loci in *TREX1*-expressing RPE-1 cells (**b**) (*n* = 3, except *HBB* site *n* = 2, biologically independent experiments) (*P* = 0.0005 for *CXCR4*, 0.0262 for *ABCA3*, 0.0029 for *FANCD2* and 0.0028 for *UROS*, respectively) and U2OS cells (**c**) (*n* = 2, biologically independent experiments) by phosphorothioate ssODN protection. Editing sites and HDR mutations are shown in Supplementary Fig. 9. Black dots indicate use of unprotected (un) ssODN templates, and blue dots represent 5′ and 3′ PT ssODN templates. Each dot represents an individual biological replicate, and bars represent the mean. All *P* values were calculated using an unpaired and two-sided *t*-test, *P < 0.05, **P < 0.01, ***P < 0.001. Source data are provided as a Source Data file. Source data

To determine whether *TREX1* expression is a predictive marker for CRISPR–Cas9-mediated HDR efficiency, we tested an integrated BFP-to-GFP reporter in Jurkat, MDA-MB-231, U2OS, HeLa and K562 cells. HeLa cells are one of the highest *TREX1*-expressing cell lines according to the Human Protein Atlas (https://www.proteinatlas.org/), and we found that they exhibit low basal CRISPR–Cas9-mediated HDR. Notably, HeLa HDR efficiency can be rescued to the same level as K562 cells by CRISPRi knockdown of *TREX1* or use of a protected ssODN (Supplementary Fig. 11). Among the cell backgrounds that we tested, only *TREX1*-low K562 cells exhibited high HDR with an unprotected ssODN (Fig. 3a). Protected ssODNs increased CRISPR–Cas9 HDR in all *TREX1*-expressing cells tested but did not further increase HDR in *TREX1*-low K562s (Fig. 3a).

We tested the effect of ssODN protection at introducing multiple mutation types (single nucleotide changes, short insertions and short deletions) at multiple endogenous loci in two different *TREX1*-expressing cells with normally low CRISPR–Cas9-mediated HDR (RPE1 and U2OS cells) (Supplementary Fig. 12)^39–41^. RPE1 and U2OS cells were edited using Cas9 RNPs and either protected or unprotected ssODNs, with editing efficiencies measured by NGS. Using unprotected ssODNs, we found relatively low HDR at almost every locus in both cell types. Phosphorothioate protection increased HDR efficiency by 1.8–3.7-fold at every locus tested in both RPE1 and U2OS (Fig. 3b,c). A protected ssODN also rescued HDR efficiency at *UROS, CXCR4* and *FANCD2* in HeLa cells, a line with among the highest *TREX1* expression in the Human Protein Atlas (Supplementary Fig. 13).

**Fig. 4:**
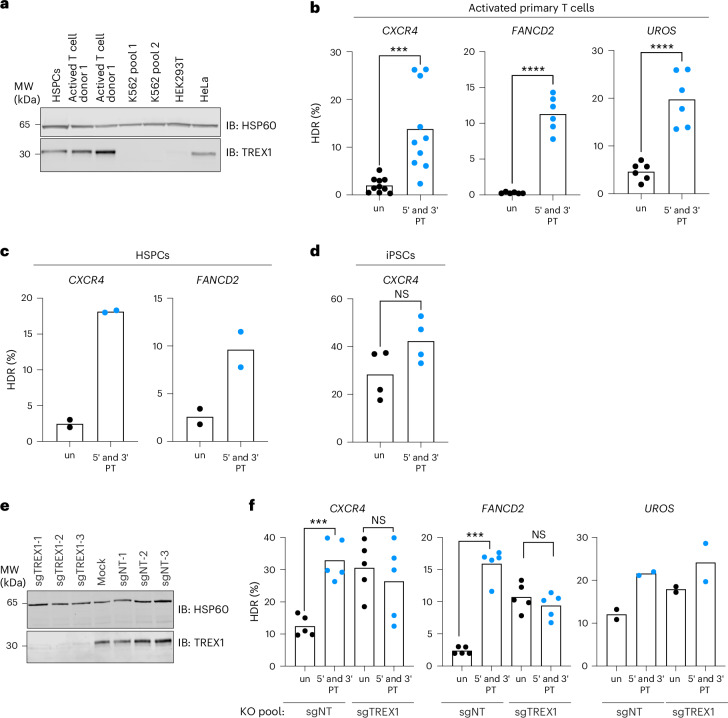
TREX1 suppresses HDR in TREX1-expressing primary cells. **a**, Primary cells, such as activated T cells and HSPCs, express TREX1. Protein extracts from the indicated primary cells and cell culture lines were analyzed using western blotting. Blots were probed with anti-TREX1 and anti-HSP60 antibodies (*n* = 2). **b**–**d**, CRISPR–Cas9-mediated HDR was improved in activated T cells and HSPCs using protected ssODN templates. HDR efficiency was measured in three different loci in activated T cells (*n* = 10, *P* = 0.0006 for *CXCR4*, *n* = 6, *P* = 6.6 × 10^−6^ for *FANCD2* and *n* = 6, *P* = 9.3 × 10^−5^ for *UROS* loci—*n* indicates the number of biologically independent experiments) (**b**), two different loci in HSPCs (*n* = 2, biologically independent experiments) (**c**) and a single locus in iPSCs (*n* = 4 biologically independent experiments, *P* = 0.0865) (**d**). Black dots represent measurements with unprotected (un) ssODN templates, and blue dots represent measurements with 5′ and 3′ protected (PT) ssODN templates. **e**, Protein extracts of activated T-cells from the indicated samples were analyzed using western blotting. Blots were probed with anti-TREX1 and anti-HSP60 antibodies (*n* = 2). **f**, Knockout of *TREX1* in activated T-cells increases CRISPR–Cas9-mediated HDR from unprotected ssODN templates (*n* = 5, *P* = 2.8 × 10^−5^, *P* = 0.3815 for *CXCR4* and *P* = 6.1 × 10^−8^, *P* = 0.1103 for *FANCD2*, *n* = 2 for *UROS*). Black dots represent measurements with unprotected (un) ssODN templates, and blue dots represent measurements with 5′ and 3′ protected (PT) ssODN templates. Each dot represents an individual biological replicate, and bars represent the mean. All *P* values were calculated using an unpaired and two-sided *t*-test:  < 0.05, **P < 0.01, ***P < 0.001. Source data are provided as a Source Data file. NT, non-targeting; IB, immunoblotting; KO, knockout; NS, not significant. Source data

Next, we investigated whether TREX1 is expressed in primary human cells, such as hematopoietic stem and progenitor cells (HSPCs), CD33-activated T cells and induced pluripotent stem cells (iPSCs). We performed western blot analysis on whole-cell extracts obtained from primary human cells as well as cell lines, including K562, HEK293 and HeLa. Consistent with the qRT–PCR results mentioned earlier, K562 and HEK293 cells showed minimal expression of TREX1 (Fig. 4a). Similarly, we observed no detectable TREX1 expression in iPSCs. However, TREX1 protein was detected in HSPCs, activated T cell and HeLa cell extracts (Fig. 4a).

Based on the expression pattern of TREX1, we hypothesized that using protected ssODNs would enhance CRISPR–Cas9-mediated HDR rates in HSPCs and T cells but not in iPSCs. Indeed, the use of protected ssODNs significantly increased HDR efficiencies in activated T cells at the three different loci we tested (ranging from a 4.3-fold increase in *UROS* editing to a 56-fold increase in *FANCD2* editing) (Fig. 4b). Furthermore, we observed substantial increases in HDR levels (7.2-fold and 3.7-fold increase, respectively) at two different loci in HSPCs (Fig. 4c). Consistent with their lack of TREX1 expression, gene editing in iPSCs showed a minimal HDR increase (1.5-fold) when protected oligos were employed (Fig. 4d), further confirming the role of TREX1 as a regulator of CRISPR–Cas9-mediated HDR in human primary cells. We also investigated the effect of TREX1 in TREX1^+^ mouse breast cancer cell lines, including 4T1 and E0771 LMB and the mouse colorectal cancer cell line CT26. In each case, the use of protected ssODNs increased HDR efficiency in the BFP-to-GFP reporter system (Supplementary Fig. 14), indicating that the restrictive role of TREX1 for HDR extends beyond human cell contexts.

To test whether the improved HDR that we observed in primary cells with protected ssODNs was related to TREX1 expression, we established *TREX1* knockout pools in activated T cells using three non-targeting gRNAs or three gRNAs targeting *TREX1* (Fig. 4e). In these T cell pools, we then performed a second edit using either unprotected or protected ssODNs. At three separate loci, knockout of *TREX1* increased HDR from unprotected ssODN templates so that they performed similarly to protected ssODN templates (Fig. 4f). These data further indicate that TREX1 restricts CRISPR–Cas9-mediated ssODN HDR in human primary cells.

Phosphorothioate protection did not allow the use of shorter donor homology arms (for example, by preventing degradation) (Supplementary Fig. 15, top). However, this result is convoluted with a critical role for long homology arms in gene conversion during endogenous HDR^42^. Additionally, we examined the use of the DNA-PKcs inhibitor AZD7648 (ref. ^43^) in combination with protected oligos in HeLa cells. AZD7648 further increased HDR rates beyond that of donor protection alone (Supplementary Fig. 15, bottom), suggesting that avoidance of TREX1 to increase donor abundance can be multiplexed with other HDR improvement strategies.

### HDR donor type affects TREX1 activity

So far, our data have primarily focused on the impact of TREX1 on ssODN templates. However, many other DNA donor types are used during genome editing. We, therefore, asked whether TREX1 also destabilizes circular and linear double-stranded DNA (dsDNA) and recombinant adeno-associated virus (rAAV) donors that are commonly used for inserting large DNA cargoes^44^.

First, we conducted gene editing experiments using plasmid dsDNA donors in TREX1-suppressed or wild-type HeLa cells. We observed a significantly lower starting HDR efficiency when plasmid DNA templates were employed^45^. Consistent with an exonuclease function of TREX1, HDR efficiency was not significantly affected upon TREX1 suppression when using these covalently closed plasmid templates (Supplementary Fig. 16).

**Fig. 5:**
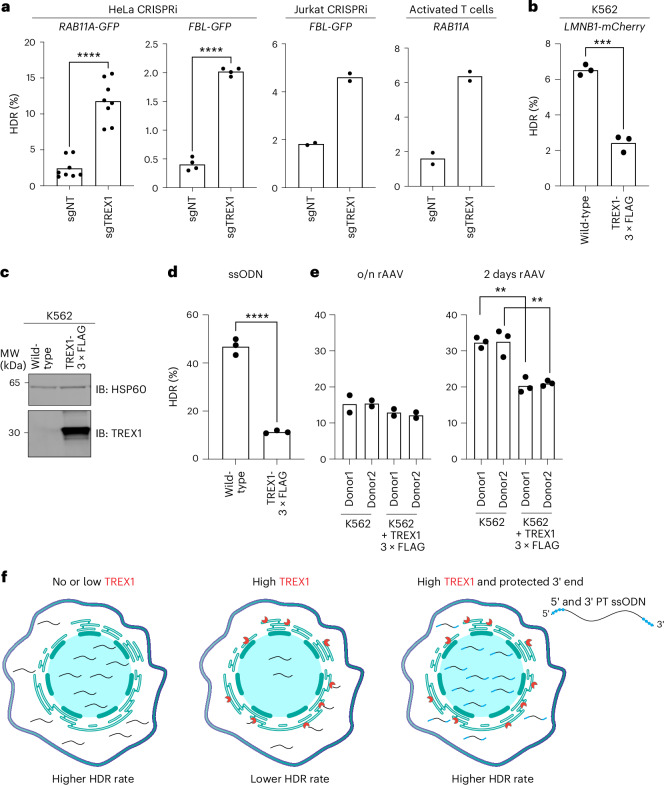
TREX1 has differential activity on various types of HDR donors. **a**, CRISPRi suppression or pooled CRISPR knockout increases HDR efficiency from linear dsDNA templates in multiple cell backgrounds. GFP was introduced into *RAB11A* and *FBL* loci in HeLa cells (*n* = 8, *P* = 1.1 × 10^−6^ for *RAB11A* and *n* = 4, *P* = 2.4 × 10^−7^ for *FBL*, respectively), activated T cells (from Fig. 4e, with *RAB11A* only) and Jurkat cells (*FBL* only) (*n* = 2). Each dot represents an individual biological replicate measurement. **b**, Overexpression of TREX1 in K562 cells reduces the efficiency of linear dsDNA-templated HDR of mCherry into the *LMNB1* locus (*n* = 3, *P* = 0.0002). Each dot represents an individual biological replicate measurement. **c**, Western blot shows the overexpression of the TREX1-3×FLAG construct in K562 cells. Blots were probed with anti-TREX1 and anti-HSP60 antibodies (*n* = 1). **d**, TREX1 overexpression significantly decreases CRISPR–Cas9-mediated HDR in a BFP-to-GFP assay when using an ssODN template (*n* = 3, *P* = 5.4 × 10^−5^). Each dot represents an individual biological replicate measurement. **e**, TREX1 overexpression only moderately represses rAAV-templated HDR in a BFP-to-GFP assay. rAAV donor templates carrying promoterless GFP were used in K562 wild-type and TREX1-overexpressed cells. After CRISPR–Cas9 targeting with the gRNA, rAAV was incubated with the cells overnight (*n* = 2) or for 2 d (*n* = 3, *P* = 0.013), and HDR was measured by the presence of GFP-positive cells in FACS 5 d after nucleofection. Each dot represents an individual biological replicate measurement. **f**, Overall model of TREX1’s role in repressing CRISPR–Cas9-mediated HDR. In TREX1-low cells, ssODN templates are abundant throughout the cell and available in the nucleus for efficient CRISPR–Cas9-mediated HDR. In cases where TREX1 expression is high, ssODN templates are degraded 3′ to 5′ by TREX1 at the endoplasmic reticulum. This reduces ssODN availability in the nucleus and decreases HDR efficiency. Phosphorothioate protection prevents TREX1 digestion of the ssODN, maintaining a high template concentration throughout the cell and enabling efficient CRISPR–Cas9-mediated HDR. Each dot represents an individual biological replicate, and bars represent the mean. All *P* values were calculated using an unpaired and two-sided *t*-test: *P < 0.05, **P < 0.01, ***P < 0.001. Source data are provided as a Source Data file. NT, non-targeting; IB, immunoblotting; o/n, overnight. Source data

We then examined whether linear PCR-amplified dsDNA donors coding for templated insertion of GFP or mCherry into various endogenous genomic loci were affected by TREX1 expression^39^. We found that suppressing TREX1 resulted in higher editing efficiency (4.8-fold and 5.1-fold increase, respectively) when inserting GFP into the *RAB11A* and *FBL* loci in HeLa cells (Fig. 5a). Notably, the linear *RAB11A* donor responsive to TREX1 expression was otherwise identical to the covalently closed plasmid *RAB11A* donor that was unresponsive. We also observed that repressing *TREX1* increased linear dsDNA-templated HDR in Jurkat cells when inserting GFP into the *FBL* locus as well as in activated T cells when inserting GFP into the *RAB11A* locus (Fig. 5a).

To further ensure that these effects were dependent on TREX1 expression, we overexpressed a TREX1-3×FLAG cDNA construct in K562 TREX1-null cells and tested the effect upon linear dsDNA-based HDR. In wild-type K562 cells, we saw approximately 6.5 ± 0.3% mCherry^+^ cells when inserting mCherry at *LMNB1*. However, TREX1 overexpression substantially reduced the efficiency of HDR to 2.4 ± 0.47%, further supporting the notion that TREX1 expression is causative of HDR effect when DNA donors have free 3′ ends (Fig. 5b).

Lastly, we investigated whether the levels of TREX1 affect HDR editing when using rAAV donors. We employed two different AAV6 HDR donors carrying a promoterless eGFP sequence to serve as donor templates for our BFP-to-GFP conversion assay. Because AAV6 donors are compatible with blood cells^46^, we conducted the experiment in K562 TREX1-null cells and compared these to TREX1-3×FLAG-overexpressing cells (Fig. 5c). Overexpression of TREX1 was sufficient to greatly reduce the efficiency of HDR from an ssODN template, as expected (Fig. 5d). Overnight exposure of cells to two different AAV donors was not much affected by TREX1 levels (Fig. 5e). However, it has been suggested that AAVs deliver their DNA cargo into the nucleus, which theoretically should be protected from ER-localized TREX1 (refs. ^47,48^). Furthermore, AAVs have extensive terminal hairpins in their inverted terminal repeats (ITRs), which might protect them from TREX1’s exonuclease activity. We, therefore, lengthened the timeframe of the experiment and found that HDR was reduced in TREX1-expressing cells after 2 d of AAV exposure.

Taken together, our data strongly indicate that *TREX1* is a major restrictor of CRISPR–Cas9-mediated HDR in human cell lines and primary cells. TREX1 activity is most potent on linear, unprotected DNA donor molecules than on covalently closed or molecules where the 3′ end is less accessible. Chemical protection of the 3′ end of even linear and exposed DNA templates is sufficient to circumvent TREX1 activity (Fig. 5f).

## Discussion

We identified TREX1 as an exonuclease that restricts CRISPR–Cas9-mediated HDR in human cell lines. Cell lines expressing low levels of TREX1 are competent to perform HDR and have become the cell backgrounds in which much work using CRISPR–Cas9 HDR has been done. By contrast, FA cells and multiple immortalized cell lines express high levels of TREX1 and are compromised in their ability to perform HDR. Removal of TREX1 from these cells markedly increases CRISPR–Cas9 HDR, as does the use of chemically protected ssODN templates that would specifically escape TREX1 activity. The presence of TREX1 as the sole hit during screening and the ability of TREX1 removal alone to rescue HDR to high levels suggests that, in cells normally transiting through the cell cycle, it may be a dominant factor involved in restricting CRISPR–Cas9 HDR from exogenously provided templates. Certain stresses, including innate immune receptor activation, have been shown to induce TREX1 expression^49,50^. These stresses might also reduce HDR efficiency in contexts where it is normally active.

Phenomenological studies previously hinted at the benefits of shielding the ends of HDR templates during genome editing. Dual 5′ and 3′ phosphorothioate protection of an ssODN can increase HDR in some contexts^51,52^. However, a lack of mechanistic understanding meant that the ideal situations to protect with phosphorothioates were unknown, and they have not been generally adopted by the field. ssODNs can also be protected by integrating Cas9 binding sites at their termini or by circularizing the single-stranded template^53,54^. We propose that all of these protections at least partly work by protecting against TREX1 activity, and their benefits would be even further enhanced in cell backgrounds with high levels of TREX1. Very long ssODNs (1+ kb) might enhance gene editing efficiencies by adding a length buffer against TREX1 exonuclease activity. However, generating the large quantities of long ssODNs required for efficient HDR can be challenging, and their use is not yet widespread.

We suggest that *TREX1* expression is a biomarker for the use of ssODN protection, which adds rational choice to the empiric and exhaustative process of designing genome editing HDR experiments. We furthermore found that the dual-end protection previously employed is dispensible, and 3′-end protection is sufficient for fast, inexpensive improvements to HDR in contexts with appreciable TREX1 expression. The knowledge-driven application of ssODN protection parallels the application of targeted cancer therapies in appropriate genetic backgrounds and mirrors the recent discovery that endogenous La protein promotes prime editing by protecting prime editing guide RNAs (pegRNAs)^55^. Ongoing searches for TREX1 inhibitors to treat cancer might yield a small-molecule inhbitior of TREX1 that could be useful during genome editing workflows, in case phosphorothioate-protected templates are toxic to a certain cell type^56^.

Overall, our data shed mechanistic light on why donor template protection increases HDR, provide a concrete biomarker for the targeted use of template protection and resolve longstanding confusion around why some cell types are easier to edit than others.

## Methods

### Cell lines

Healthy donor, *FANCA*^*–/–*^ (FA55) and *FANCD2*^*–/–*^ (FA-75) LCLs were generously gifted by Paula Rio (CIEMAT). K562, Jurkat, U2OS, MD-MBA-431, RPE-1 hTERT and HeLa cells were obtained from the American Type Culture Collection or the Berkeley Cell Culture facility. The RPE-1 hTERT *TREX1*^*−/−*^ cell line was used in this study^32^. LCLs were cultured in RPMI 1640 medium (GlutaMAX, from Thermo Fisher Scientific, 61870010) supplemented with 20% Gibco FBS (Thermo Fisher Scientific, 10270106), 1% Gibco penicillin–streptomycin (P/S) solution (Thermo Fisher Scientific, 15140122), 0.005 mM Gibco β-mercaptoethanol (Thermo Fisher Scientific, 31350010) and 1% Gibco MEM non-essential amino acids (Thermo Fisher Scientific, 11140050). K562 and Jurkat cells were cultured in RPMI 1640 GlutaMAX media supplemented with 10% FBS and 1% P/S solution. U2OS, MD-MBA-421, RPE-1 hTERT and HeLa cells were cultured in DMEM, high glucose, GlutaMAX pyruvate medium (Thermo Fisher Scientific, 10569010) supplemented with 10% FBS and 1% P/S. All cells were cultured at 37 °C with 5% CO_2_ in a humidified incubator. Cell lines were regularly tested for mycoplasma with a MycoAlert Mycoplasma Detection Kit (Lonza, LT07-318).

To generate CRISPRi cell lines, we packaged a CRISPRi (pHR-EF1a–dCas9–HA–mCherry–KRAB–NLS) construct into lentiviruses in HEK293T cells. The lentiviral supernatant was filtered and used to transduce LCLs and HeLa cells. After transduction, mCherry^+^ cells were sorted using an SH800 Cell sorter (Sony). Because LCLs failed to survive as single cells in 96-well plates, we first seeded approximately 500 mCherry^−^ LCLs per well in 96-well plates and then sorted single mCherry^+^ cells into 500 mCherry^−^ cells to overcome the viability problem. mCherry^+^ LCLs were further enriched with consecutive rounds of sorting until reaching mCherry purity higher than 90%.

For retroviral transduction of 3×flag-TREX1-wt in RPE-1 hTERT *TREX1*^−/−^ cells, open reading frames were cloned into pQCXIZ, which confers resistance to zeocin. Constructs were transfected into Phoenix amphotropic packaging cells using calcium phosphate precipitation. Cell supernatants containing retrovirus were filtered, mixed 1:1 with target cell media and supplemented with 4 μg ml^−1^ polybrene. Successfully transduced cells were selected using zeocin (Life Technologies).

#### Primary cell culture

CD34^+^ HSPCs were cultured in SC media (SFEMII and CC110 (STEMCELL Techonologies)). CD4^+^ T cells were purified from frozen human peripheral blood Leukopak (STEMCELL Techonologies) by negative selection using an EasySep Human T Cell Enrichment Kit (STEMCELL Technologies) according to the manufacturer’s instructions and cryopreserved in CryoStor CS5 (STEMCELL Technologies). Purified T cells were cultured in X-VIVO 15 Media (Lonza) supplemented with 5% human AB serum (GeminiBio) and 100 IU ml^−1^ human IL-2 (Miltenyi Biotec). For gene editing experiments, T cells were activated using TransAct (Miltenyi Biotec) according to the manufacturer’s instructions. For Fig. 4e,f, T cells were activated 1 d after thaw using CD3/CD28 Dynabeads (Thermo Fisher Scientific) according to the manufacturer’s instructions. The beads were removed after 2 d. The T cells were used in gene editing experiments on either day 3 or day 4. iPSCs were cultured in complete StemFlex media (Gibco Life Technologies) and seeded in the coated plates with Synthemax (Corning).

#### Mouse cell lines

4T1 and CT26 cell lines were cultured in RPMI media (supplemented with 10% FBS and 1% P/S solution). E0771.LMB cells were cultured in DME HG media (supplemented with 10% FBS, 5% HEPES and 1% P/S solution). All cells were cultured at 37 °C with 5% CO_2_ in a humidified incubator.

### In vitro transcription of gRNAs

gRNAs were in vitro transcribed as described (10.17504/protocols.io.dwr7d5)^57^. In brief, overlapping oligomers, indicated in Supplementary Table 2, containing a T7 promoter, protospacer and gRNA scaffold, were amplified by Q5 High-Fidelity DNA Polymerase (New England Biolabs, M0491L) for 15 cycles. Then, 1 µM T7FwdLong and 1 µM T7RevLong were used as a template and amplified by T7FwdAmp and T7RevAmp in 50-µl reaction volume. Next, 8 µl of PCR-amplified product was used for the in vitro transcription using an NEB HiScribe T7 High Yield RNA Synthesis Kit (New England Biolabs, E2040S), incubating at 37 °C for 18 h in the thermocycler. Then, the reaction was supplemented with DNase I (Qiagen, 79256) for 30 min at 37 °C, followed by Quick CIP (New England Biolabs, M0525S) treatment for 1 h at 37 °C. The gRNAs were later purified with an miRNeasy kit (Qiagen, 217604), and concentration was measured by Qubit RNA Broad Range assay (Thermo Fisher Scientific, Q10211) and stored at −80 °C.

### Genome-wide library construction

To shuttle genome-wide sgRNAs, we first amplified the cassettes including sgRNA using the primer set priEK-35 and priEK-37 from the CRISPRi-V2 library (Addgene, 1000000093) using Phusion polymerase (New England Biolabs, M0530L) under the following condition: 30 s at 98 °C, then 15 cycles of 15 s at 98 °C, 15 s at 53 °C, 15 s at 72 °C and then a final extension for 10 min at 72 °C. Amplified PCR fragments were digested overnight with Bpu1102I (BlpI) (Thermo Fisher Scientific, ER0091) and BstXI (Thermo Fisher Scientific, ER1021) at 37 °C. The digested DNA fragments were separated in a 10% TBE gel to cut the DNA band corresponding (~33 base pairs (bp)). The gel pieces were crushed by spinning for 3 min at 20,000*g* and then eluted in water at 37 °C overnight. DNA later was precipitated with the NaOAc/EtOH method. In addition, we linearized the vector carrying mutated GFP sequence with the same restriction enzymes, Bpu1102I (BlpI) and BstXI, for 4 h at 37 °C. The linearized DNA product was separated by 0.8% agarose gel electrophoresis and excised from the gel. DNA was cleaned using a QIAquick Gel Extraction Kit (Qiagen). The DNA further was cleaned with the NaOAc/EtOH method. For the ligation reaction, 500 ng of linearized vector and 1.9 ng of insert were incubated with T4 DNA Ligase (New England Biolabs, M0202L) for 16 h at 16 °C. The ligated plasmids were purified by isopropanol/5 M NaCl precipitation and resuspend in 13 µl of elution buffer. Then, 1 µl or 2 µl of the purified ligation reaction was mixed with 25 µl of MegaX DH10B T1^R^ electrocompetent cells (Thermo Fisher Scientific, C640003) and recovered in S.O.C medium for 1.5 h. The bacteria were plated in 24.5 × 24.5-cm LB agar plates containing ampicillin resistance. While plating the bacteria in 24.5 × 24.5 cm, the dilutions of bacteria were performed as well to detect approximate coverage of the sgRNA library. Grown colonies were collected by scraping from LB agar plates. Plasmids were recovered by several midi-preps (Qiagen Plasmid Plus Midi Kit).

Quality of the library was determined by NGS. Sequencing libraries were prepared by amplifying sgRNA cassettes with the primers priEK_i5-1 and priEK_i7-1 and secondary PCR to put the sequencing adapters priEK_501 and priEK_701 (indicated in Supplementary Table 2). The reaction was sequenced by MiSeq, and sgRNA distribution of the cloned library was analyzed using custom scripts.

### Production of sgRNA library lentiviruses

To produce lentivirus from the sgRNA library, approximately 7 million HEK293T cells were seeded in a 15-cm plate in 20 ml of DMEM medium with 10% FBS and 1% P/S. The next day, HEK293T cells were transfected with the library. Per plate, in a 5-ml tube, 15 µg of sgRNA library, 12 µg of delta VPR and 3 µg of VSVG were resuspended with 1.3 ml of Opti-MEM and mixed with 270 µl of polycation polyethylenimine (PEI) 1 mg ml^−1^ (1 µl to 3 µg of DNA). The mix was incubated at room temperature for 20 min and then added on top of HEK293T cells in a drop-wise manner. The media were changed on the following day. The virus-containing cell culture media were collected 48 h and 72 h after the transfection. The viral media were combined and filtered using a 0.45-µm PES membrane (Thermo Fisher Scientific, 295-3345), aliquoted into 15-ml Eppendorf tubes, snap frozen and stored at −80 °C.

### CRISPR screen

*FANCA*^*–/–*^ CRISPRi cells were grown to 150 million cells before transduction with the genome-wide CRISPRi library. Because LCLs were extremely difficult to transduce with lentiviruses, *FANCA*^*–/–*^ CRISPRi cells were directly resuspended in virus-containing media and seeded in six-well plates in the presence of 8 µg ml^−1^ polybrene. The coverage determined by BFP^+^ cells was around 300× per sgRNA. Twenty-four hours after transduction, cells were collected and transferred to T75 flasks. A day later, gRNA-containing cells were selected with puromycin treatment (0.5 µg ml^−1^) for 96 h. At this moment, BFP^+^ cells were over 90%. Upon puromycin selection achieved, cells were split into two replicates and were maintained for 250× coverage throughout the screen. Twenty days after transduction, cells were subjected to Ficoll gradient (Ficoll Paque Plus, Millipore Sigma, GE17-1440-02). In total, 1 × 10^6^ cells were electroporated with 400 pmol SpCas9 nuclear localization sequence (NLS), 480 pmol L2 gRNA targeting BFP and 500 pmol BFP-to-GFP ssODN template using CM-189 and SF solution (Lonza, 4D electroporator) per replicate. Cells were further expanded in culture before sorting. Before the sort, a background population was collected for downstream NGS analysis. The sort was performed in two steps: first, thresholding was set to enrich the GFP^+^ population from approximately 0.5% to 70%, and then a stringent sort was performed to achieve approximately 99% pure GFP^+^ cells. Cells were pelleted and stored at −80 °C until genomic DNA (gDNA) extraction.

### NGS sample preparation and screen analysis

gDNA was extracted using a Gentra Puregene Cell Kit (Qiagen, 158912) gDNA extraction protocol. In brief, for the background samples (from a total of 25 million cells), the cell pellet was resuspended in 3 ml of cell lysis solution and then mixed with 15 µl of RNase A solution at 37 °C for 20 min and cooled down for 10 min on ice, and then 1 ml of protein precipitation buffer was added. The mix was vortexed thoroughly and spun down for 10 min at 2,000*g*. The gDNA-containing supernatant was mixed with 100% isopropanol (3 ml) by inverting the 15-ml tube for 50 times. The gDNA was pelleted by centrifugation at 2,000*g* for 5 min and then washed with 70% EtOH. After removing 70% EtOH, gDNA was resuspended in 200 µl of hybridization buffer and incubated at 50 °C for 1 h. DNA amount was measured by NanoDrop. For the GFP^+^ cells, the protocol was adjusted for the low cell number. In the gDNA precipitation step, glycogen was added to facilitate the gDNA precipitation.

Purified gDNA was used for the further PCR amplification of sgRNA cassettes following the protocol (https://weissman.wi.mit.edu/resources/IlluminaSequencingSamplePrep.pdf). For the background samples, 5 µg of gDNA per reaction was used. For the sort background samples, 30 PCR reactions were performed and combined later. Because we had very limited DNA from GFP^+^ cells, we used 1-µg amount of DNA per reaction and performed two reactions. The PCR products were purified by two rounds of Sera-Mag magnetic beads (Cytiva, 29343957). The concentrations were measured by a Qubit 1× dsDNA High Sensitivity Assay (Thermo Fisher Scientific, Q33232), and the samples were pooled according to their anticipated read counts. The samples were sequenced on a NextSeq 2000.

Screening data were analyzed using the standard protocols in MaGECK (version 0.5.9.5) and drugZ. MaGECK was used to get the gRNA counts per each sgRNA in the population by using the following command:

mageck count -l CRISPRi_v2_humantop5.library.txt–fastq 20210427A-EK1_R1.fastq.gz 20210427A-EK2_R1.fastq.gz 20210427A-EK3_R1.fastq.gz 20210427A-EK4_R1.fastq.gz 20210427A-EK5_R1.fastq.gz 20210427A-EK6_R1.fastq.gz–sample-label bgsort_rep1,bgsort_rep2,bg_rep1,bg_rep2,gfp_rep1,gfp_rep2 -n count_mageck.txt

drugZ was used to integrate multiple guides into gene-level phenotypes relative to the background unsorted population (normZ score and FDR values)^23,24^ by using the command:


python drugz.py -i count_mageck.txt -o drugz-gfpposvsbgsort.txt -c bgsort_rep1,bgsort_rep2 -x gfp_rep1,gfp_rep2


(Supplementary Table 1).

ggplot in R was used to produce the graph in Fig. 1b by using the following code:


drugz_gfp_sort <- read_tsv("~/Documents/FALCLScreen_results/drugZ_output/drugz-gfpposvsbgsort.txt")


ggplot(drugz_gfp_sort, aes(y = normZ, x = rank_synth, size = -log10(fdr_supp)))+


 geom_jitter(alpha = 0.4)+ theme_classic()ggsave("drugZ_v2.pdf",width = 20, height = 10, units = c("cm"))


Further modifications were made using Adobe Ilustrator.

### RNP electroporation for BFP-to-GFP reporter assay and genomic loci targeting

RNP electroporation was performed as described (10.17504/protocols.io.dm649d)^9^. In brief, 36 pmol sgRNA and 30 pmol SpCas9-NLS were mixed in Cas9 buffer (20 mM HEPES at pH 7.5, 150 mM KCl, 1 mM MgCl_2_, 10% glycerol and 1 mM tris (2-carboxyethyl) phosphine (TCEP) reducing agent). The mixture was incubated at room temperature for 20 min. Then, 1 × 10^5^ to 2 × 10^5^ cells were collected and spun down at 300*g* for 5 min. The cell pellets were resuspended in 15 µl of nucleofection buffer (Lonza). Then, 5 µl of RNP mixture was added to the cell suspension with 0.3 µl of 100 µM (30 pmol) ssODN (BFP-to-GFP template) template. Five days after electroporation, cells were collected and subjected to flow cytometry with an Attune Flow Cytometer (Thermo Fisher Scientific). Downstream analysis was performed using FlowJo version 10.8.2 software.

Mouse cells were electroporated following the same protocol as other cells (see above) using the supplemented SE buffer provided by Lonza and using the CM-150 program.

For endogenous locus targeting, 100 pmol SpCas9-NLS was mixed with 120 pmol gRNA in Cas9 buffer, and the mixture was incubated for 20–30 min at room temperature or 37 °C. In total, 1 × 10^5^ to 2 × 10^5^ cells were collected and resuspended in 15 µl of nucleofection buffer (Lonza). For each reaction, 100 pmol ssODN was then added before nucleofections. Electroporations were performed in the strip format, with 20-µl volume of cells and RNP mix. The following kit and program for each cell type was selected: K-562 (SF kit/FF-120), RPE-1 (P3 kit/EA-104), U2OS (SE kit/CM-130), MDA-MB-231 (SE kit/CM-130), HeLa (SE kit/CM-130) and Jurkat (SE kit/CL-120). After electroporation, pre-warmed 80 µl of DMEM or RPMI medium was added into strips. Cells were incubated in the hood for 10 min and then transferred to the plates and returned to 37 °C.

ssODNs were purchased from Integrated DNA Technologies as Ultramer DNA oligos. To protect ssODNs, they were ordered with four phosphorothioate modifications at the 5′ and/or 3′ ends. To amplify PCR templates, we performed with primers for the corresponding genes following the PCR protocol: 30 s at 98 °C, then 15 cycles of 15 s at 98 °C, 15 s at 60 °C, 15 s at 72 °C and then a final extension for 10 min at 72 °C. We later used Sera-Mag beads to clean and concentrate the PCR products around 700–1,000 ng µl^−1^ and used 1,000 ng of template per nucleofection. Oligo sequence information can be found in Supplementary Table 2.

For the primary cell nucleofection, the protocol described above was used with the following changes. For HSPCs, cells were electroporated with P3 kit, ER-100 or DS-130 program. Activated T cells were electroporated with P3 kit and CN-114 program, and iPSCs were electroporated with P3 kit and CB-150 program. After electroporation, cells were returned to their corresponding media, as indicated above.

For rAAV donors, custom-made ssAAV vectors with serotype 6 were prepared and sent to the viral vector facility (VVF) of the Neuroscience Center of Zurich (ZNZ) to generate viral particles. AAVs were ultracentrifuged (OptiPrep) and dial-filtered in 1× PBS pH 7.4, 1 mM MgCl_2_ and 2.5 mM KCl. The titers of the donors were determined as 7.9 × 10^12^ vector genomes per milliliter (vg/ml) and 8.1 × 10^12^ vg/ml, respectively. Approximately 10^10^ viral particles for 1 × 10^5^ cells were added 5 min after nucleofection. The media were changed either after overnight or 2 d after transduction.

### gDNA extraction

Cell pellets were collected 72–96 h after electroporation and resuspended in QuickExtract solution (Lucigen, QE09050) and subjected to gDNA extraction while incubating for 10 min at 65 °C, 5 min at 98 °C and then holding at 4 °C. After the incubation, 1 µl of gDNA was taken for further PCR reactions for NGS.

### NGS

Primers containing adaptor binding sites (indicated in Supplementary Table 2) were designed to amplify 150–200 bp around the cut sites. First, gDNA was amplified using NEBNext Ultra II Q5 Master Mix for 30 cycles and then cleaned with SPRI beads (SeraMag Select (Cytiva, 29343052) or in house). From the purified reactions, around 10–20 ng of DNA was used as input for the second PCR reaction to add i7/i5 indexes for the samples in nine reaction cycles. Amplicons were then purified again with 0.8× SeraMag beads, and samples for the same genomic loci were combined. The amplicon length and purity were analyzed by a TapeStation with D1000 DNA flow cells (Agilent). Pools were combined based on their amount and desired read number (50,000–100,000 reads per sample). The combined samples were sequenced in Illumina sequencers (MiSeq or NextSeq 2000) in the Genome Engineering and Measurement laboratory at ETH Zurich.

### NGS analysis

The sequencing reads were demultiplexed and analyzed with CRISPresso2 (version 2.0.20b) in batch mode^58^ with default parameters other than minimum average read quality (- q) of 30 and minimum single bp quality of (- s) 10 and the quantification window (- w) 20. Reads with a frequency lower than 0.5% were disregarded before further analysis. Results were then normalized to sum up to 100%.

### Immunoprecipitation–western blot

Cells indicated in Figs. 2, 4 and 5 were harvested by trypsinization and lysed in RIPA buffer (5 mM Tris-HCl pH 7.6, 150 mM NaCl, 1% NP-40, 1% sodium deoxycholate, 0.1% SDS), supplemented with phosphatase inhibitors (10 mM NaF, 20 mM β-glycerophosphate) and protease inhibitor (Thermo Fisher Scientific) at approximately 10^6^ cells per milliliter and incubated on ice for 20 min. Lysates were sonicated with a Bioruptor Plus sonication device (Diagenode) for 15 cycles ON/OFF (high, 4 °C). Sonicated lysates were incubated on ice for 20 min and centrifuged at 16,000*g* and 4 °C for 20 min, and supernatants were transferred into French tubes before protein quantification using the Pierce BCA protein assay (Thermo Fisher Scientific). Lysate equivalent to 10–50 µg of proteins was mixed to 1× Laemmli buffer (50 mM Tris, 10% glycerol, 2% SDS, 0.01% bromophenol blue, 2.5% β-mercaptoethanol), resolved by SDS-PAGE (Life Technologies) and transferred to nitrocellulose membranes (Amersham). Membranes were blocked in 5% milk in TBS with 0.1% Tween 20 (TBS-T) and incubated with primary antibody overnight at 4 °C, washed three times in TBS-T and incubated for 1 h at room temperature with HRP-conjugated secondary antibody. After three washes in TBS-T, imaging was performed using enhanced chemiluminescence (Thermo Fisher Scientific).

### Antibodies

Primary antibodies included: anti-TREX1 (1:1,000 dilution) (Abcam, ab185228), anti-β-actin (1:2,000 dilution) (Abcam, ab8224), anti-RPA32 (1:1,000 dilution) (Abcam, ab2175), anti-flag (1:2,000 dilution) (Abcam, f1804) and anti-HSP60 (1:1,000 dilution) (Santa Cruz Biotechnology, sc-1052).

Secondary antibodies (1:10,000–15,000 dilution) included: goat anti-mouse IgG HRP (Thermo Fisher Scientific, 31432), donkey anti-rabbit IgG HRP (SouthernBiotech, 6441-05), anti-rabbit secondary antibody IRDye 800 CW (LI-COR Biosciences, 926-32213) and anti-goat IRDye 800 CW (LI-COR Biosciences, 926-32214).

### Biotin-ssODN immunoprecipitation

In total, 5–6 × 10^6^ RPE-1 hTERT parental or *TREX1*^*−/−*^ cells expressing were electroporated with 5 nmol biotinylated or unprotected ssODN (Integrated DNA Technologies) in 100 µl of final volume using a Lonza 4D-Nucleofector (Methods). Two hours after electroporation, cells were harvested by trypsinization, washed with PBS and resuspended in lysis buffer (50 mM Tris pH 7.5, 200 mM NaCl, 0.075% NP-40, protease inhibitors) at 10^7^ cells per milliliter. Cells were then dounce homogenized by 10 strokes with a tight-fitting pestle. Lysates were incubated on ice for 20 min and centrifuged at 16,000*g* and 4 °C for 20 min, and then input samples were taken for immunoblotting. To reduce non-specific binding of proteins to the beads, lysates were precleared by incubation with Protein G Dynabeads (Invitrogen) for 30 min at room temperature. To pull down the biotinylated ssODN, the cleared lysate was transferred onto streptavidin Dynabeads (10 μl per sample; Invitrogen) and again incubated for 30 min at room temperature. The beads were washed eight times with lysis buffer and then eluted with 2× Laemmli buffer (100 mM Tris, 20% glycerol, 4% SDS, 0.02% bromophenol blue, 5% β-mercaptoethanol). Immunoblotting of input and eluted samples (diluted 1:2) was performed as described in the Methods.

### qRT–PCR

RNA extraction was performed using an RNeasy Mini Kit (Qiagen) according to the manufacturer’s instructions. Then, 1 μg of RNA per sample was used for reverse transcription using iScript Reverse Transcription Supermix for qRT–PCR (Bio-Rad, 1708841) according to the manufacturer’s instructions. qRT–PCR reactions were set up using SsoAdvanced Universal SYBR Green Supermix (Bio-Rad, 1725271) and run in triplicates using a QuantStudio 6 system (Thermo Fisher Scientific). A complete list of primers used in qRT–PCR can be found in Supplementary Table 2.

### Imaging of TREX1-GFP and H2B-iRFP during gene editing

In total, 700,000 RPE hTERT GFP-TREX1 H2B-iRFP cells were nucleofected with Cas9 RNP (final concentrations: sgNT (L2 sgRNA) or CXCR sgRNA 0.72 μM, Cas9 enzyme 20 μM) with or without ssODN templates (BFP ssODN or CXCR4 template ssODN, final 5 μM). The nucleofected cells were plated on poly-lysine-coated chambered coverslips (ibidi, 80807) and imaged 24 h after nucleofection.

Live-cell imaging was performed at room temperature using a Nikon Eclipse Ti2-E equipped with a CSU-W1 SoRa spinning disk super-resolution confocal system, a Borealis microadapter, a Perfect Focus 4, a motorized turret and encoded stage, a five-line laser launch (405 (100 mW), 445 (45 mW), 488 (100 mW), 561 (80 mW), 640 (75 mW)), a Prime 95B monochrome digital camera and a CFI Apo TIRF ×60/1.49 NA objective lens. Images were acquired using NIS-Elements Advanced Research Software on a Dual Xeon Imaging workstation.

All images were processed by manually isolating the most in-focus single z-slice using ImageJ software. After separating channels using the ImageJ Macro ‘Batch Split Channels’ tool, images were processed by customized CellProfiler modules, where primary nuclear masks were generated, and the perinuclear ring was defined as the nuclear periphery to serve as a proxy for cytoplasm. The nuclear intensity of both GFP-TREX1 and H2B-iRFP channels were divided by perinuclear ring intensity and log_10_ transformed to provide log_10_(ratio of nuclear/cytoplasmic GFP-TREX1 or H2B-iRFP).

To access co-localization between GFP-TREX1 and H2B-iRFP channels, both channels of each image were subjected to the ImageJ plugin JACoP with manually adjusted threshold. Pearsonʼs coefficients and Mander’s coefficients were obtained, with M2 representing the fraction of GFP-TREX1 overlapping with H2B-iRFP.

Gene editing efficiencies at *CXCR4* loci were analyzed as described above by NGS.

### Statistical analysis

Each point represents an individual biological replicate, and bars represent the mean of the replicates. All *P* values were calculated using an unpaired *t*-test, *P < 0.05, **P < 0.01, ***P < 0.001, using GraphPad Prism version 9.4.1 software.

### Reporting summary

Further information on research design is available in the Nature Portfolio Reporting Summary linked to this article.

## Online content

Any methods, additional references, Nature Portfolio reporting summaries, source data, extended data, supplementary information, acknowledgements, peer review information; details of author contributions and competing interests; and statements of data and code availability are available at 10.1038/s41587-024-02356-3.

## Supplementary information

Supplementary Information Supplementary Figs. 1–16 and uncropped images for Supplementary Figs. 3, 5 and 14.

Reporting Summary

Supplementary Table 1 Screen analysis normZ and FDR values using drugZ.

Supplementary Table 2 Oligos and primers used in the study.

Supplementary Data Source Data for supplementary figures.

## Source data

Source Data Figs. 1–5 Statistical source data/unprocessed western blots.

## Data Availability

Sequencing files for the pooled screen and endogenous genome editing in cell lines and primary cells are available in Sequence Read Archive BioProject PRJNA1117357.

## Code availability

The code used to analyze the CRISPR screen and produce the graph in Fig. 1b is available in the Methods subsection titled ‘CRISPR screen’. NGS analyses are described in the Methods subsection titled ‘NGS analysis’.
