# Supplementary material for: Removal of TREX1 activity enhances CRISPR–Cas9-mediated homologous recombination
Source: Nat Biotechnol. Author manuscript; Available in PMC 2025 Jul 25. (PMC12263433; doi:10.1038/s41587-024-02356-3)
Supplement: Supplementary Information [file NIHMS2092117-supplement-Supplementary_Information.pdf]

nature biotechnology

Supplementary information https://doi.org/10.1038/s41587-024-02356-3

Removal of TREX1 activity enhances CRISPR–Cas9-mediated homologous recombination

In the format provided by the authors and unedited

Supplementary figure 1

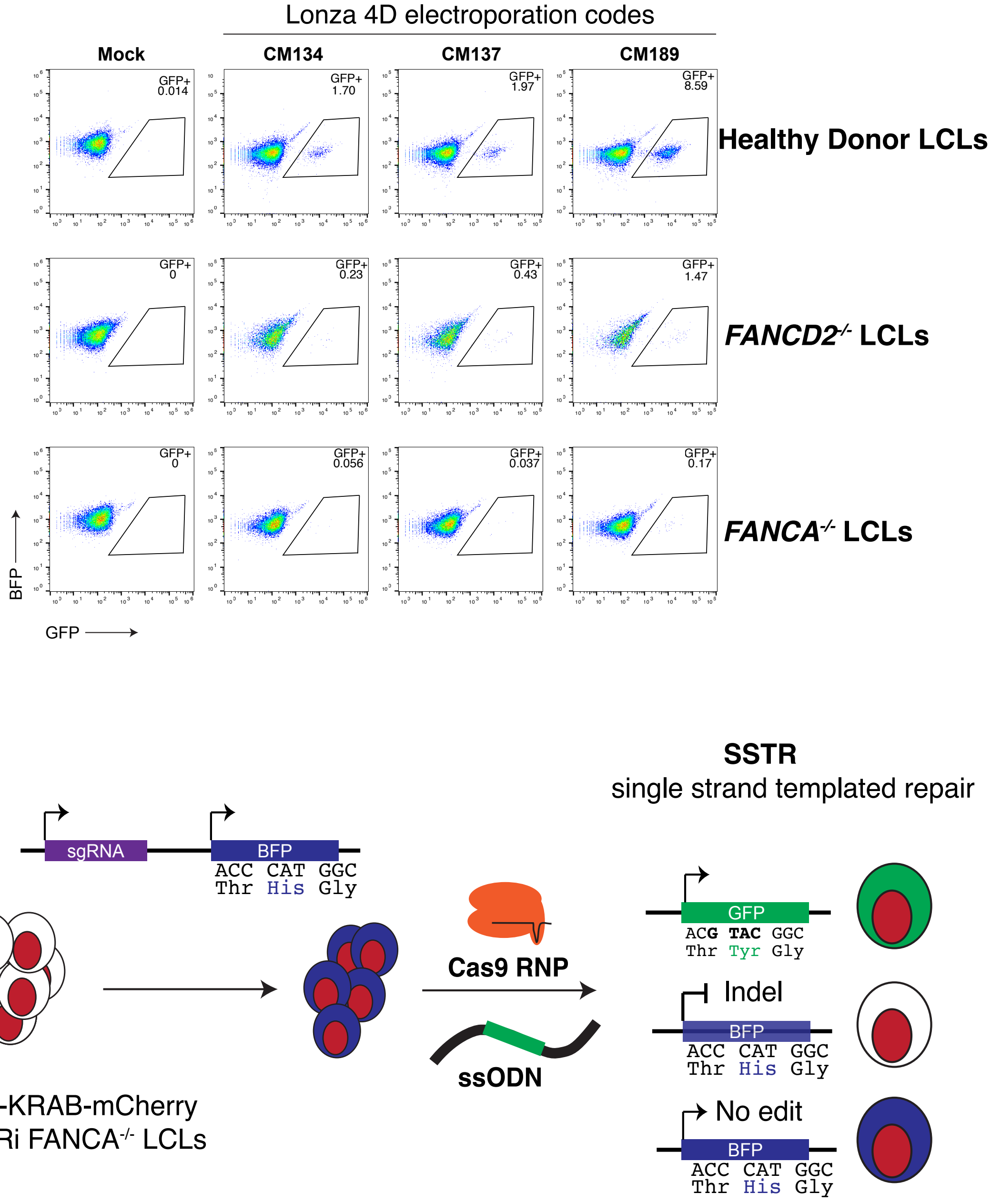

Supplementary Figure 1: Optimization of electroporation codes for healthy donor and FA patient derived LCLs.
BFP to GFP assay was performed in HD and FA LCLs. Indicated codes were tried to find the optimal targeting activity. Targeted cells were gated to measure GFP percentages 5 days after electroporation. Bottom, diagram shows genome wide screen strategy.

Supplementary figure 2

BD FACSDiva 9.0.1

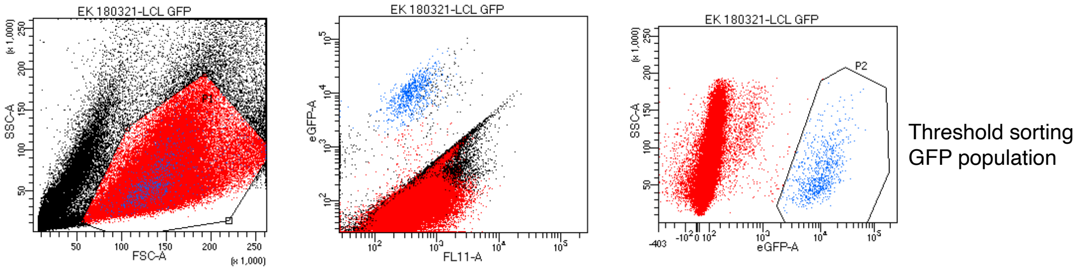

Tube: LCL GFP

Population	#Events	%Parent	%Total
All Events	200,000	####	100.0
P1	148,888	74.4	74.4
P2	814	0.5	0.4

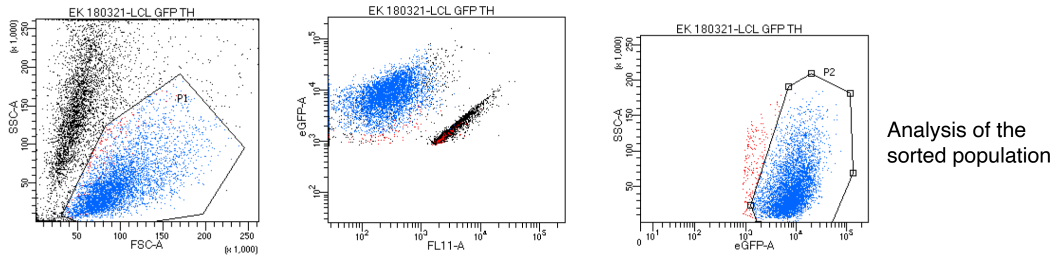

Tube: LCL GFP 2nd run

Population	#Events	%Parent	%Total
All Events	496	####	100.0
P1	269	54.2	54.2
P2	215	79.9	43.3

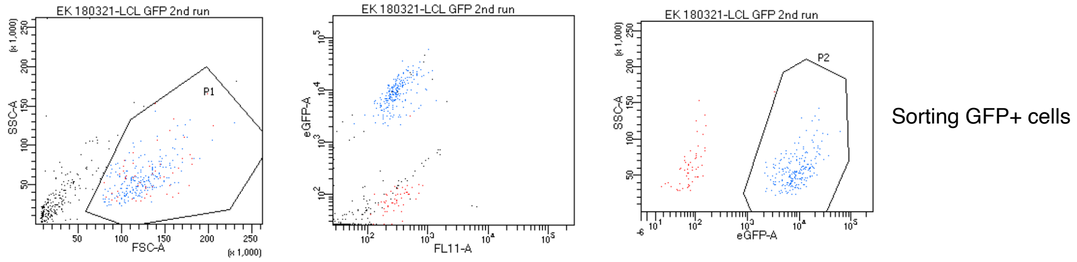

Tube: LCL GFP TH

Population	#Events	%Parent	%Total
All Events	10,000	####	100.0
P1	5,416	54.2	54.2
P2	5,230	96.6	52.3

Supplementary Figure 2: Sorting strategy for genome wide CRISPRi strategy. Representation of *FANCA*$^{-/-}$ LCLs sorting in BD FACSDiva 9.0.1. To increase GFP positive cells (originally 0.4%), threshold was set around 10^3 for the GFP gate and cells were selected according to this threshold (top panel). Later, GFP positive enriched cells (after thresholding sort around ~ 79 %) were subjected to stringent sort (middle panel). The stringent sort yielded around ~ 96 % GFP positive cells (bottom panel).

Supplementary figure 3

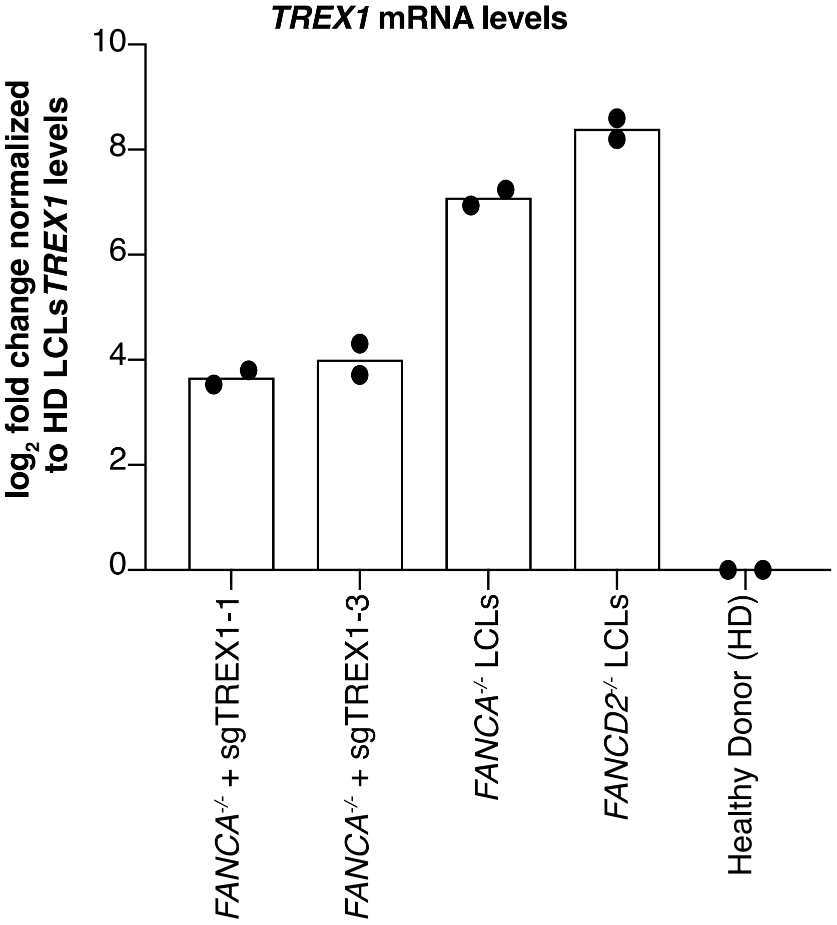

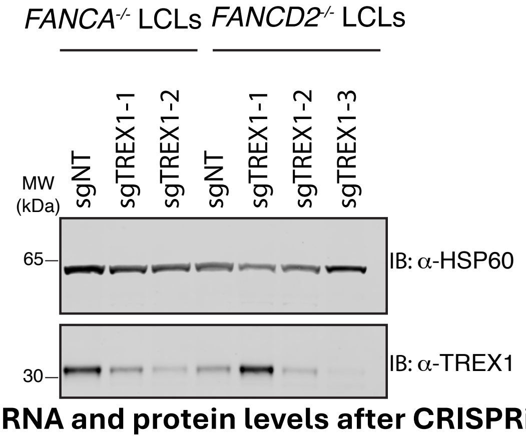

Supplementary Figure 3: *TREX1* mRNA and protein levels after CRISPRi depletion.
Top, RT-qPCR analysis of RNAs extracted from indicated cell lines. The plotted values represent the log_2 fold difference normalized to healthy donor sample. Two independent experiments were performed, as represented as dots. Bars indicate means for each cell line.
Bottom, Whole cell extracts were prepared from FANC deficient LCL cell lines. The proteins were detected by anti-TREX1, anti-HSP 60 antibodies (n = 1).

Supplementary figure 4

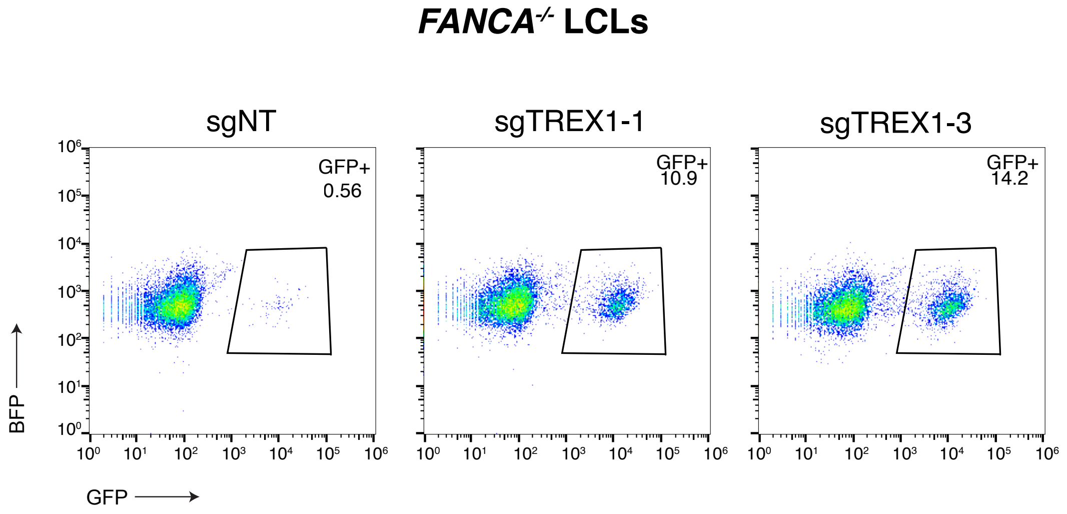

Supplementary Figure 4: Representative flow images for BFP to GFP conversion in *FANCA*$^{-/-}$ LCLs.

CRISPRi cells were transduced and selected with sgNT (non-targeting), sgTREX1-1 and sgTREX1-3 guide RNAs. Later, BFP to GFP assay was performed in these cells. 5 days after electroporation, GFP was quantified as shown in the flow graphs.

Supplementary figure 5

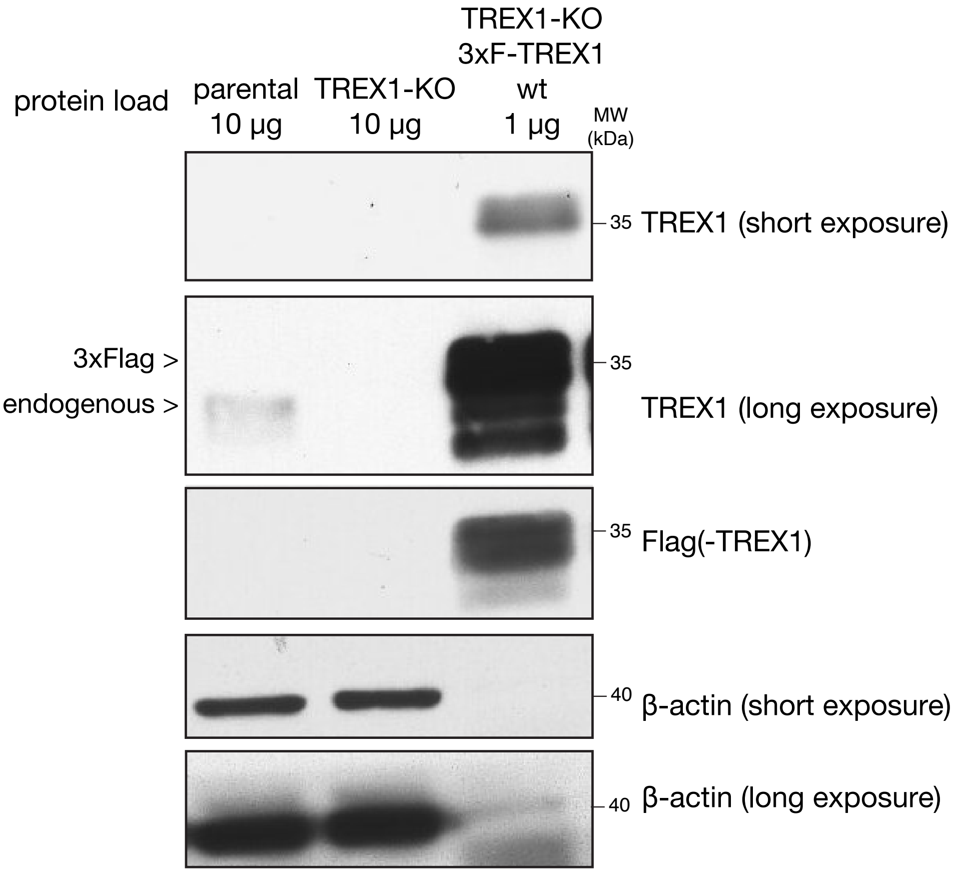

Supplementary Figure 5: Validation of re-expression of TREX1 by the Western blotting.

Whole cell extracts were prepared from RPE1 cell lines. The proteins were detected by anti-TREX1, anti-FLAG and anti-ß actin antibodies (n = 1).

Supplementary figure 6

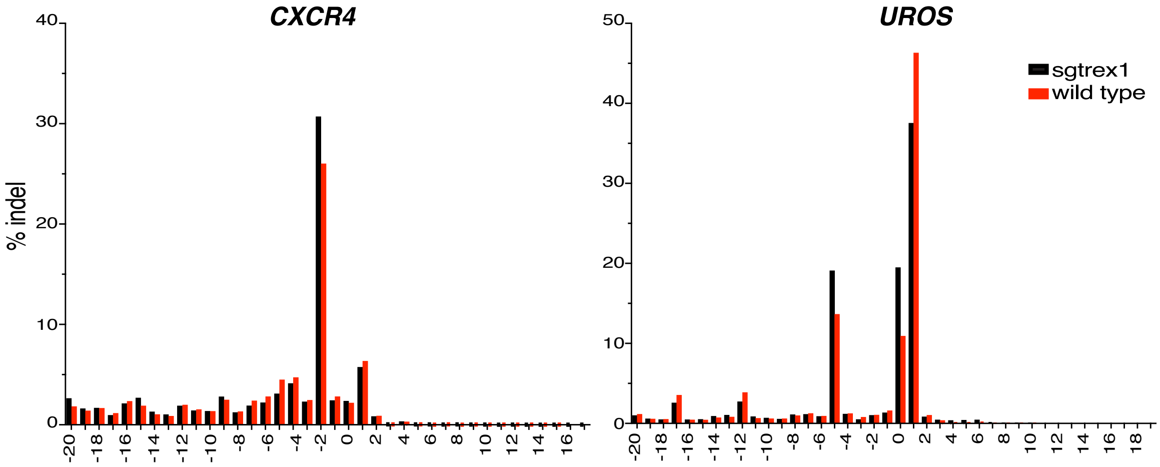

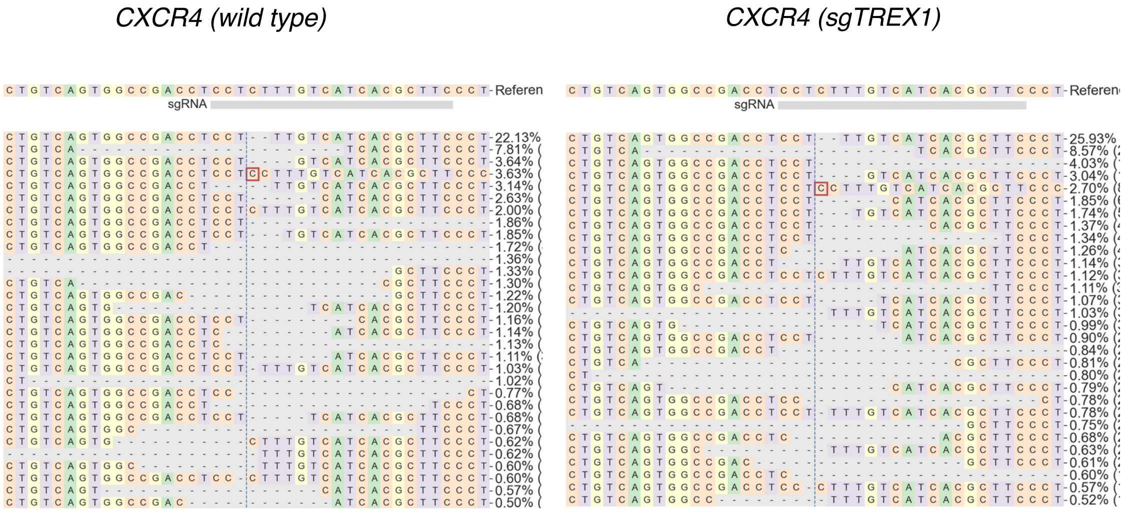

Supplementary Figure 6: Indel profiles from *CXCR4* and *UROS* loci
TREX1-suppressed or wild-type HeLa cells were targeted with CRISPR-Cas9 RNPs without ssODN templates. The indel profile around the DSB cut site, determined by CRISPResso2, was plotted for the CXCR4 and UROS loci. Black bars indicate indel levels in wild-type HeLa cells, while red bars indicate indel percentages in *TREX1*-depleted HeLa cells. The bottom panel displays the edited allele profile from the *CXCR4* locus in both wild-type and *TREX1*-depleted HeLa cells.

Supplementary figure 7

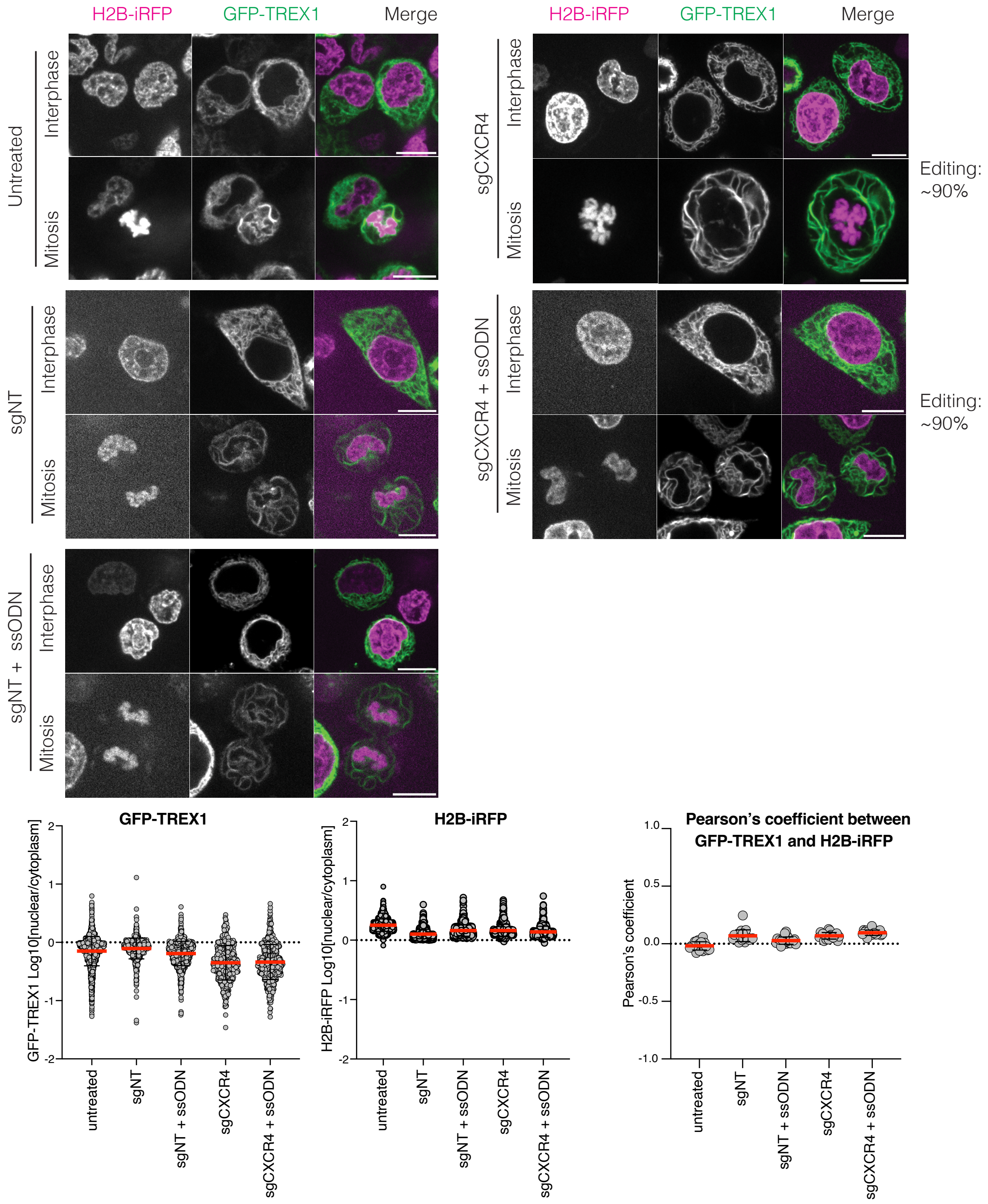

Supplementary Figure 7: GFP-TREX1 and H2B-iRFP localization after gene editing Representative images of TREX1 and H2B are shown for each condition for interphase and mitotic cell stages. The graphs indicated the analysis for the localization of nuclear vs cytoplasm for TREX1 (middle) and H2B (left). Scale bars represent 10 micron. For calculating GFP-TREX1 nuclear/cytoplasmic localization, each dot represents the individual calculation of a log10-transformed ratio of relative intensity of nuclear vs. cytosolic GFP-TREX1. A negative value means reduced TREX1 in the nucleus comparing to the cytosol, as shown in the montage. In contrast, H2B-iRFP was calculated in the

same fashion and a positive value demonstrates that H2B is majorly nuclear. The red bar represents the mean and error bars represents +/- standard deviation (SD). Pearson coefficients were calculated to measure overlap between TREX1 and H2B signal in different conditions, which shows little overlap between TREX1 and H2B, demonstrating that TREX1 is majorly cytosolic. The experiment has been repeated twice, and the data represents one biological replicate. For log10-transformed intensity ratio, more than 500 cells were analyzed for each condition. For Pearson's coefficient, 20 cells were analyzed for each condition.

Supplementary figure 8

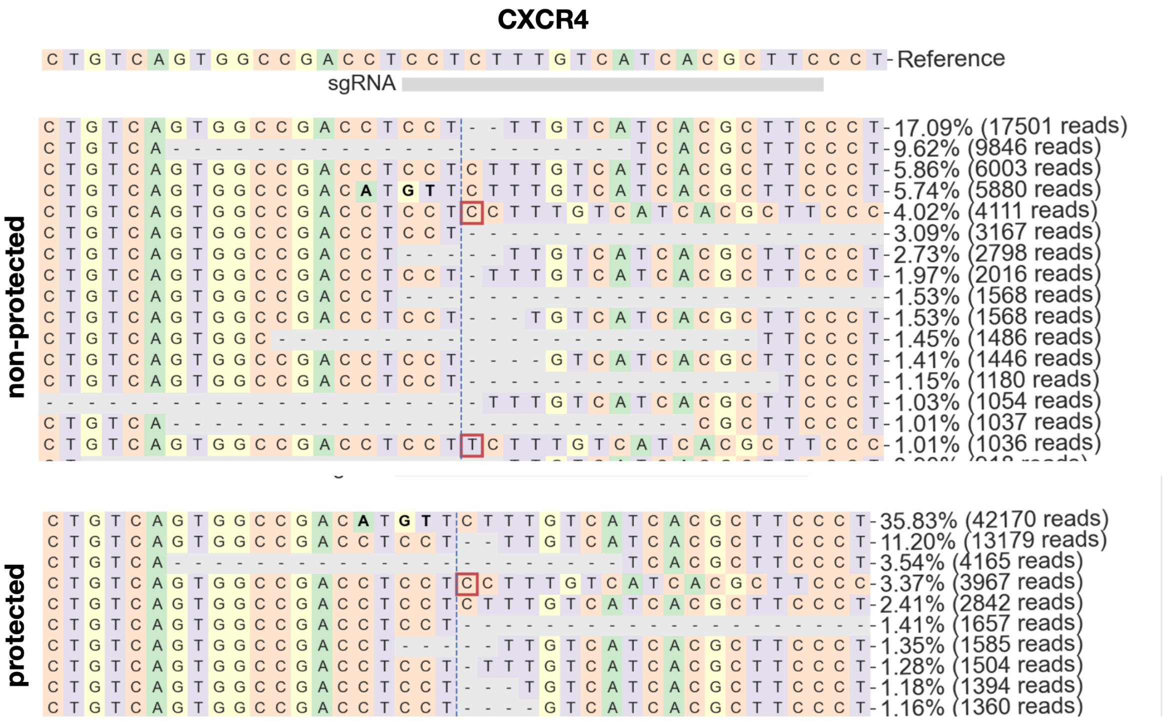

Supplementary Figure 8: Representative allele distributions in *CXCR4* locus using unprotected or protected ssODN

The allele distribution of CRISPResso2-analyzed NGS reads was visualized. The use of unprotected and protected oligos did not result in significantly different indel profiles, except for the HDR allele (3-nucleotide substitution), which notably increased when a protected oligo was used.

Supplementary figure 9

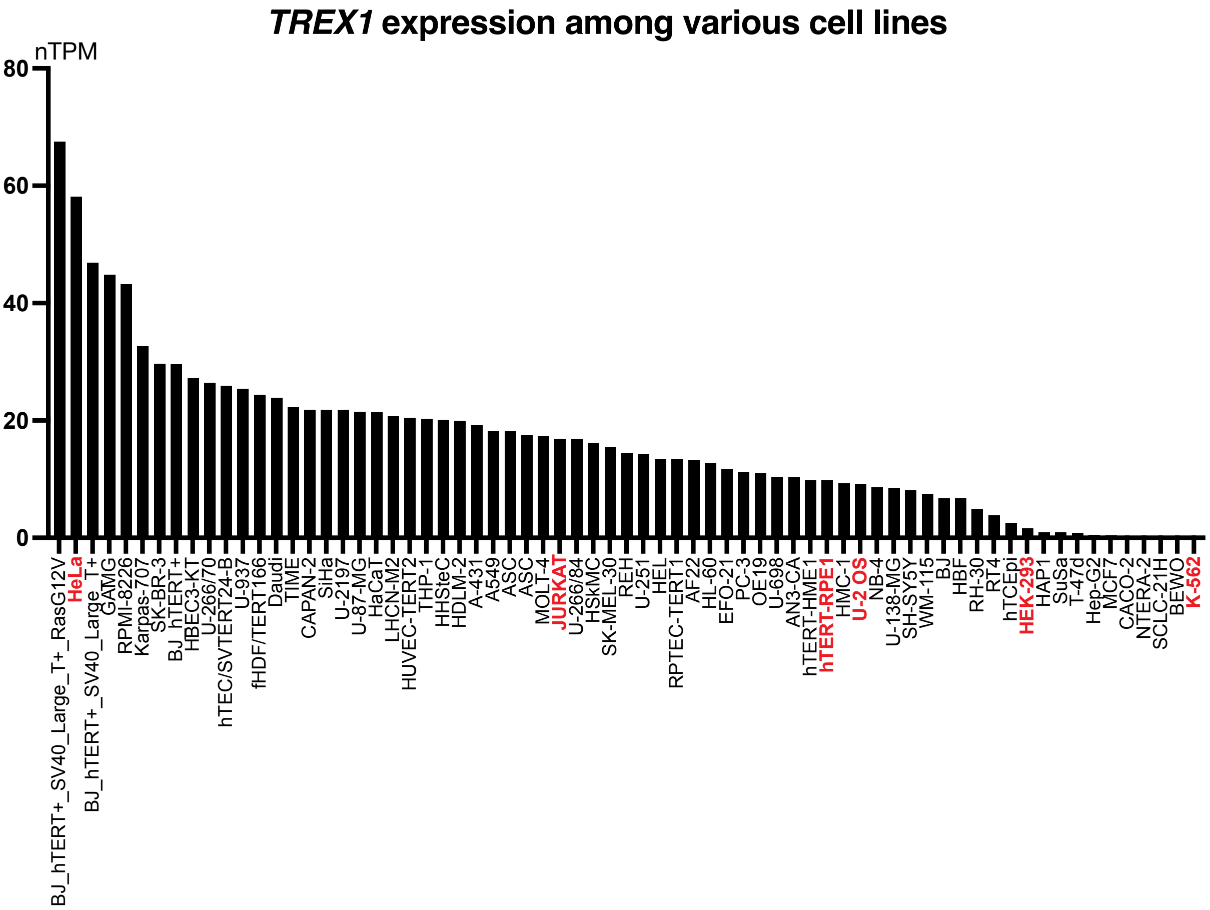

Supplementary Figure 9: *TREX1* expression dataset from Protein Atlas.
Expression of *TREX1* was searched on Protein Atlas (https://www.proteinatlas.org/) and normalized transcription expression values (nTPM) from various cell lines were replotted. Cell lines used in this study or commonly used in gene editing experiments were highlighted with red.

Supplementary figure 10

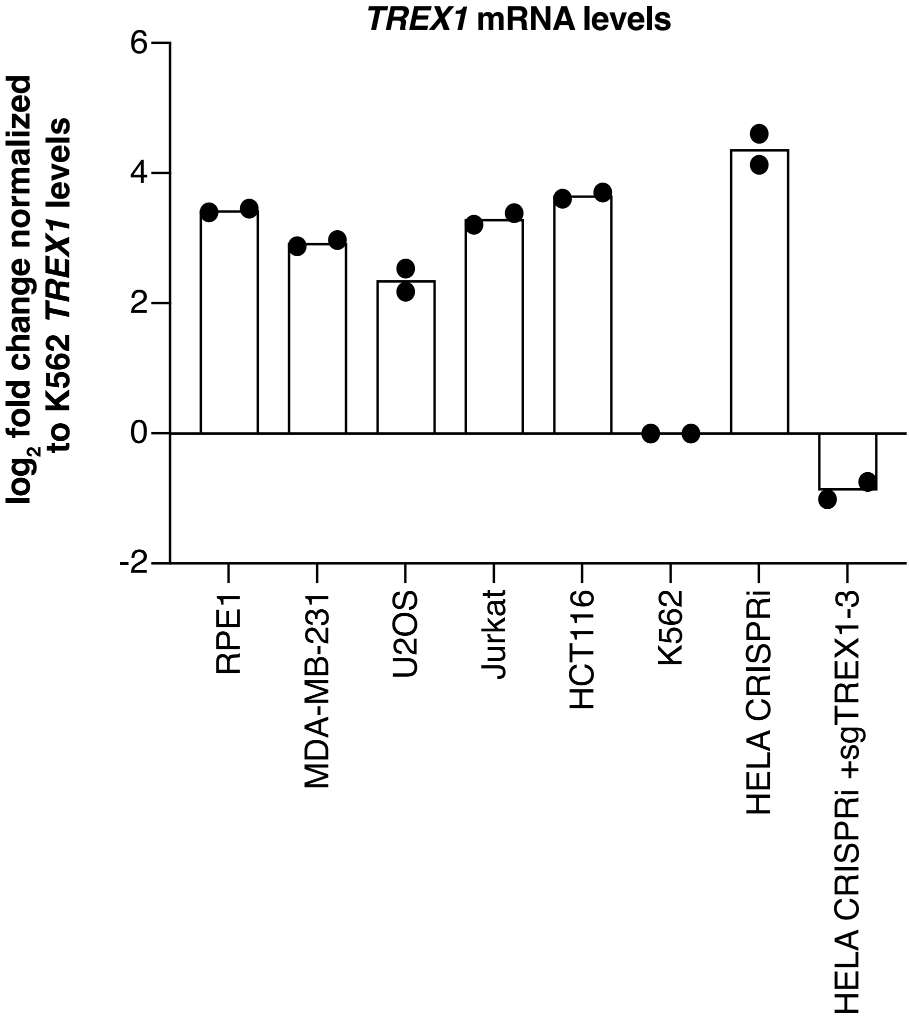

Supplementary Figure 10: RT-qPCR for *TREX1* mRNA levels in various cell lines and after CRISPRi depletion in HELA cells.

RT-qPCR analysis of RNAs extracted from indicated cell lines. The plotted values represent the log_2 fold difference normalized to K562 cell line. Two independent experiments were performed, as represented as dots. Bars indicate means of *TREX1* expression for each cell line.

Supplementary figure 11

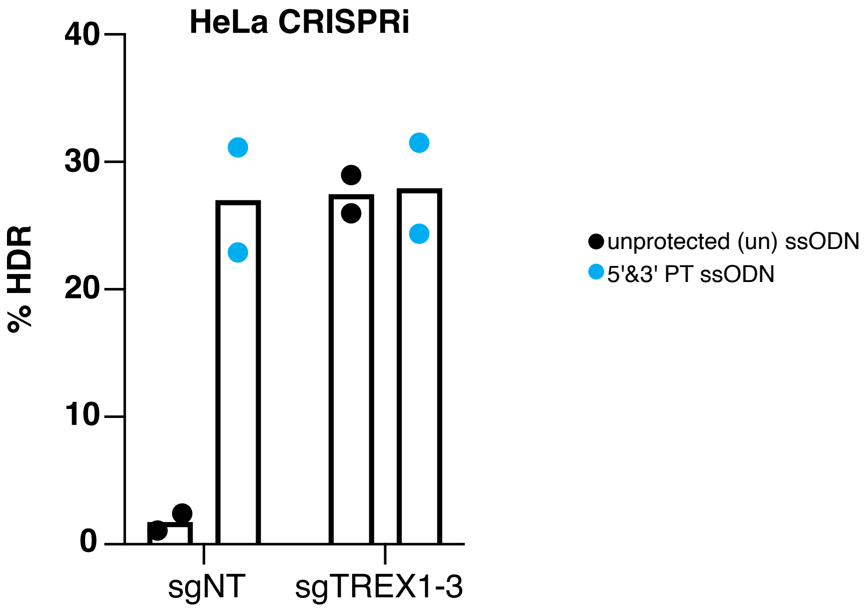

Supplementary Figure 11: Depletion of *TREX1* or using protected ssODN rescue HDR efficiency in HELA cells.
CRISPRi cell lines were transduced with sgNT (non-targeting) or sgTREX1-3 guide RNAs as indicated. BFP to GFP assay was performed with or without protected ssODN. 5 days after electroporation, GFP was measured and plotted. Two independent experiments were performed (represented as dots).

Supplementary figure 12

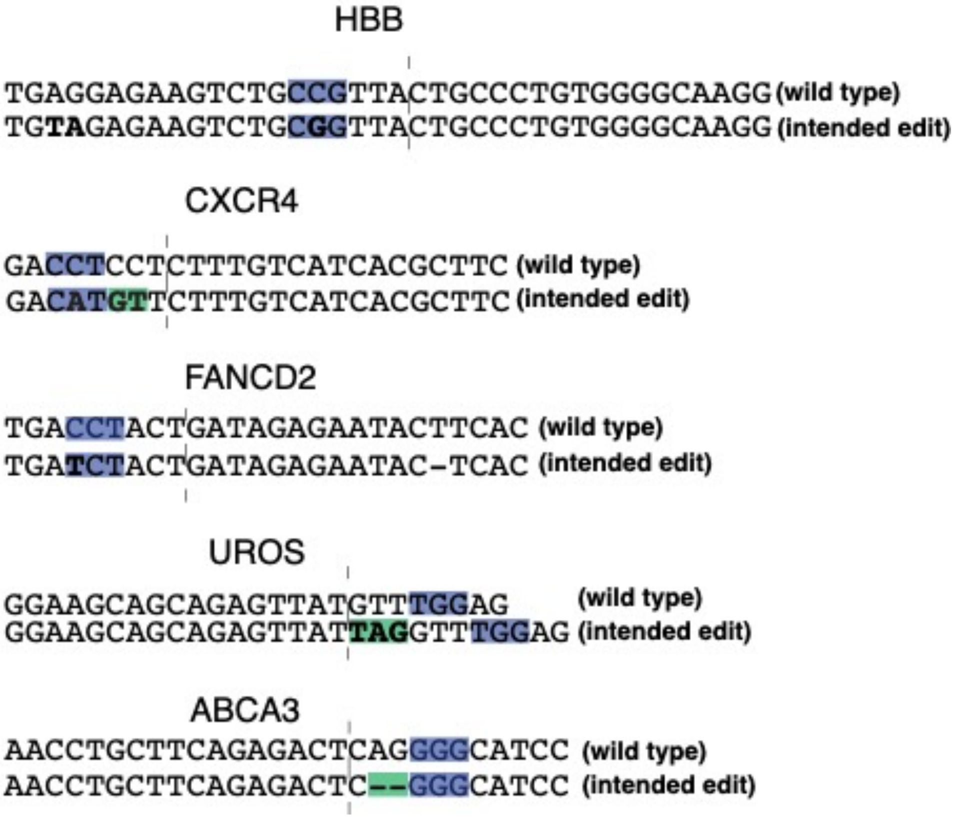

Supplementary Figure 12: Endogenous targets and the intended edits for each target.

For *HBB*, *CXCR4*, *FANCD2*, *UROS* and *ABCA3*, PAM sites are highlighted with dark blue and dashed lines show Cas9 cut site. In the intended edit site, nucleotide changes were highlighted as bold characters, deletions with –. Briefly, *HBB* intended edit is nucleotide substitutions, *CXCR4* and *UROS* intended edits are small inserts, *FANCD2* intended edit is a nucleotide substitution and deletion, *ABCA3* intended edit is two nucleotide small deletion. For *HBB, CXCR4, FANCD2,* the PAM sequences are mutated to prevent for Cas9 recutting.

Supplementary figure 13

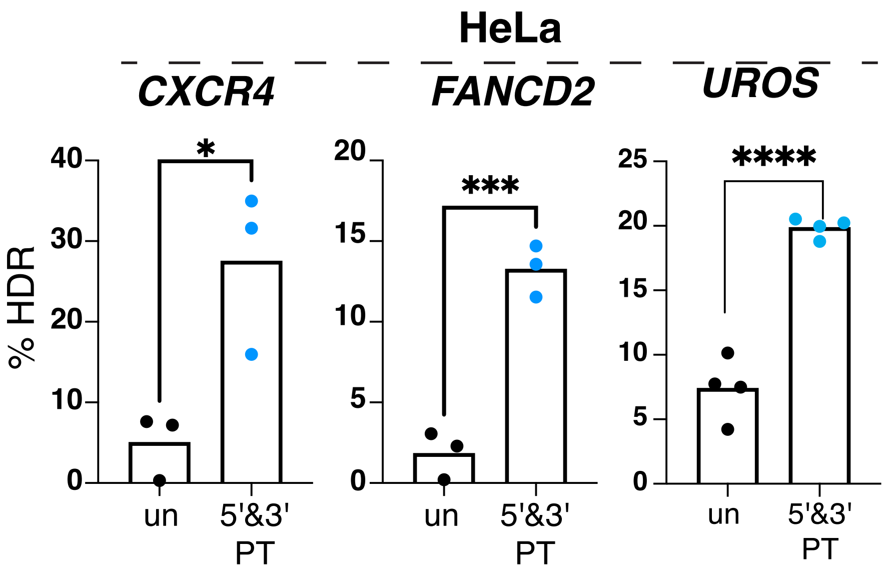

Supplementary Figure 13: *CXCR4, FANCD2,* and *UROS* targeting in HELA cell line. *CXCR4* (n = 3, *P* = 0.0236), *FANCD2* (n = 3, *P* = 0.0008), *UROS* (n = 4, *P* = 5.9×10^{-5}) genomic sites were chosen to edit by CRISPR-Cas9 and ssODN templates in HELA cells. Black dots represent the outcome of gene editing with unprotected ssODN templates and light blue dots represents the gene editing outcomes with 5'&3' PT ssODN templates. Each dot (n) represents an independent experiment. All p-values were calculated using un-paired and two-sided t test, * p<0.05, **p<0.01, ***p<0.001.

Supplementary figure 14

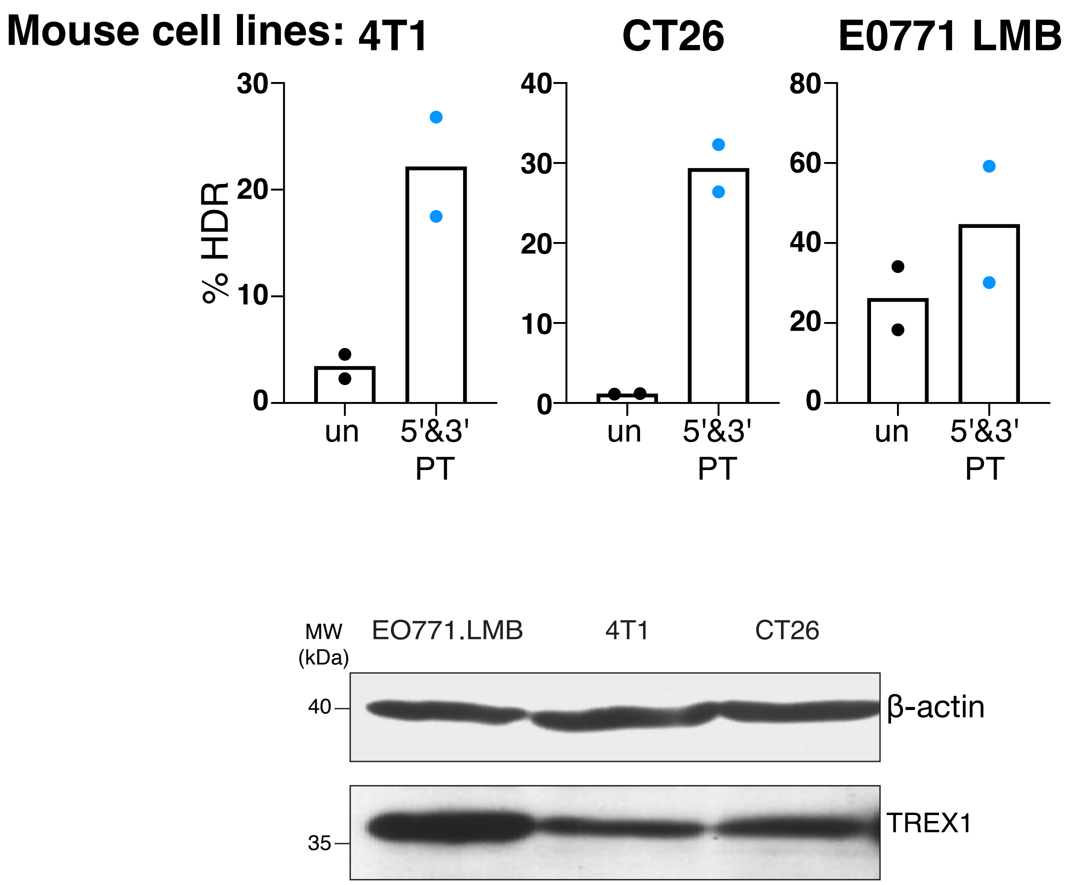

Supplementary Figure 14: BFP to GFP targeting assay in mouse cell lines
Top: The indicated mouse cell lines were transduced with the BFP reporter and subjected to CRISPR-mediated HDR, as described previously.
Bottom: Protein extracts were analyzed by western blotting, and the blot was probed with anti-beta Actin and anti-TREX1 antibodies. Each dot on the graph represents an independent experiment.

Supplementary figure 15

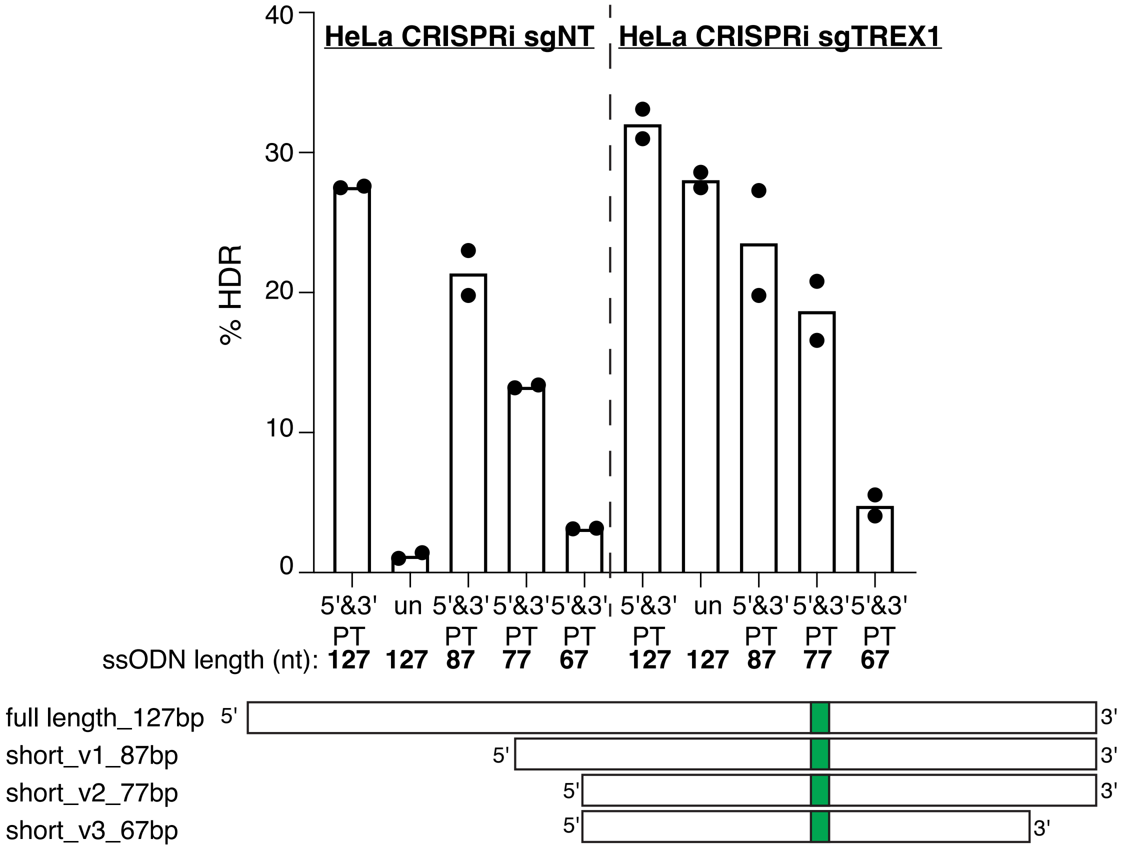

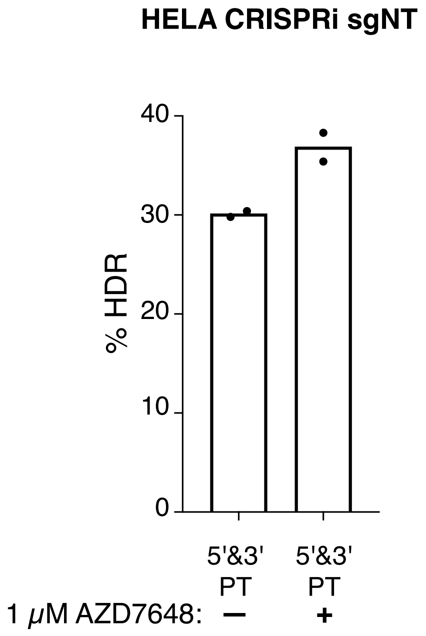

Supplementary Figure 15: BFP to GFP targeting assay with shorter homology arms in HeLa cells

Modified ssODN templates were generated, and a BFP to GFP assay was performed in TREX1-depleted HeLa cells and wild-type HeLa cells. Additionally, a BFP to GFP assay was performed in the presence of AZD7646 in wild-type HeLa cells using protected ssODN. Each dot on the graph represents an independent experiment (n = 2).

Supplementary figure 16

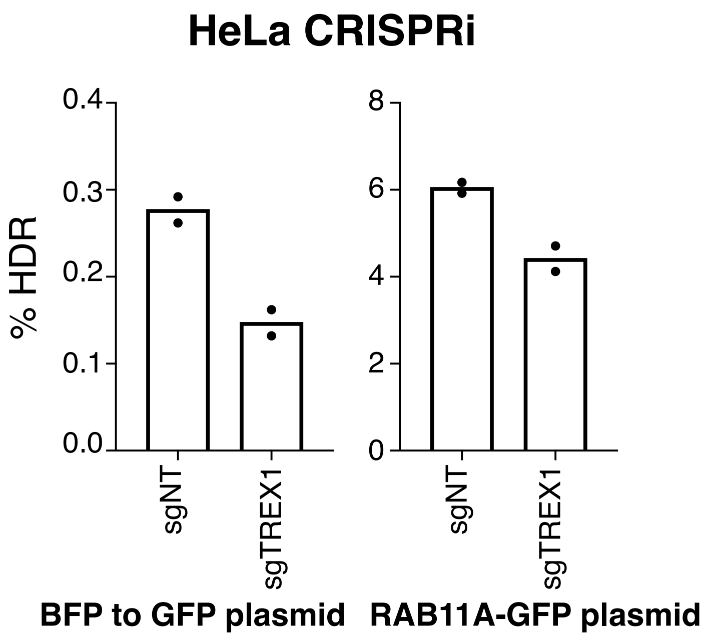

Supplementary Figure 16: HDR assay using plasmid DNA donors in HeLA TREX1 positive and negative cells.
Two different plasmid DNA donors (BFP to GFP assay donor plasmid, RAB11A-GFP plasmid) were used in CRISPR-Cas9 mediated HDR experiment (n = 2).

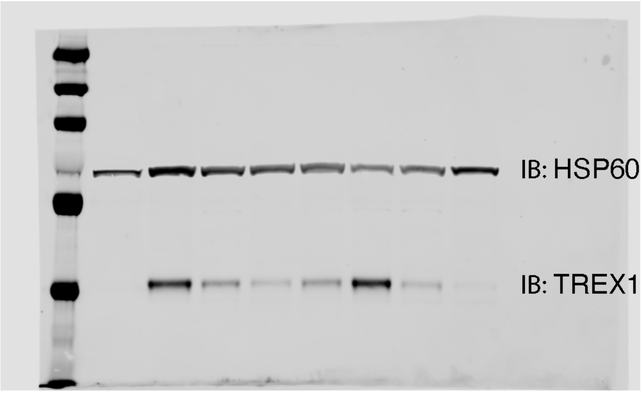

The unprocessed western blot of **Supplementary Fig 3**.

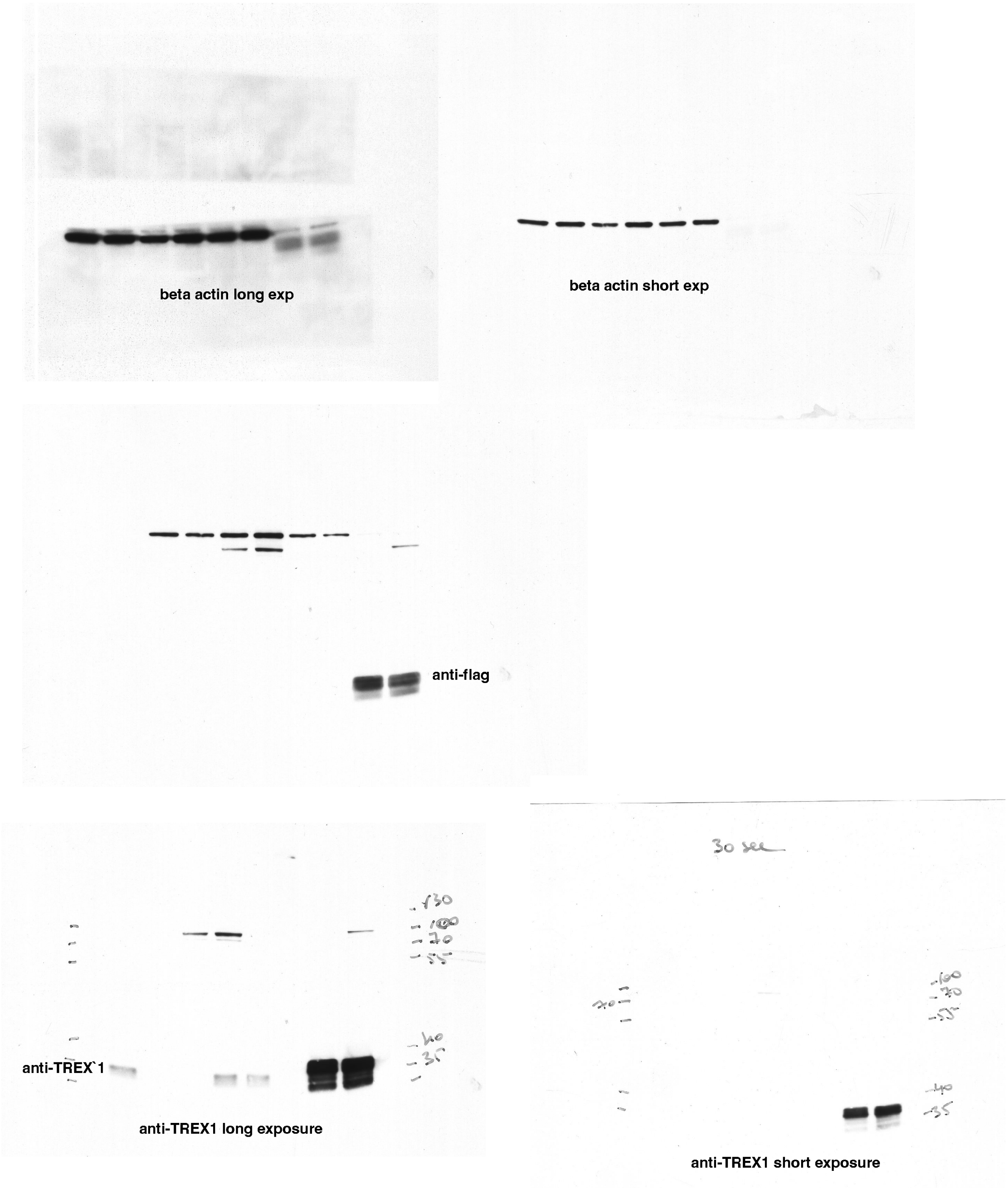

The unprocessed western blots of **Supplementary Fig 5**.

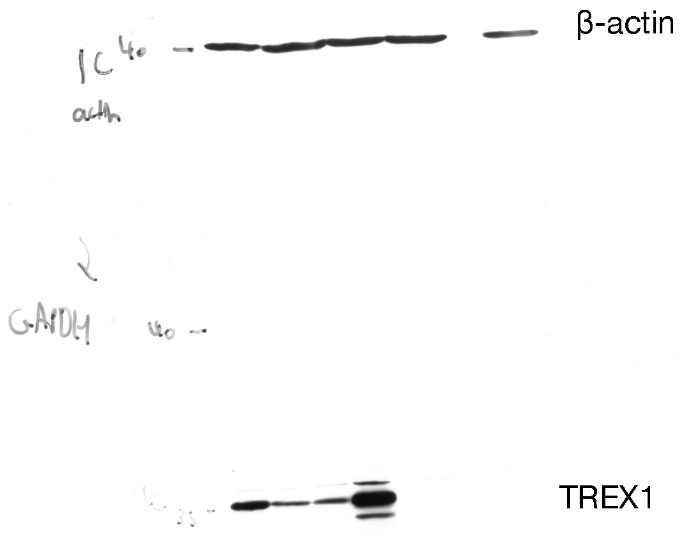

The unprocessed western blots of **Supplementary Fig 14**.
