# Supplementary material for: Removal of TREX1 activity enhances CRISPR–Cas9-mediated homologous recombination
Source: Nat Biotechnol. 2024 Aug 12;43(7):1168–76. doi: 10.1038/s41587-024-02356-3 (PMC12263433; doi:10.1038/s41587-024-02356-3)
Supplement: Supplementary file 1 [file 41587_2024_2356_MOESM2_ESM.pdf]

nature portfolio

Corresponding author(s): Jacob E. Corn

Last updated by author(s): Jul 12, 2024

Reporting Summary

Nature Portfolio wishes to improve the reproducibility of the work that we publish. This form provides structure for consistency and transparency in reporting. For further information on Nature Portfolio policies, see our Editorial Policies and the Editorial Policy Checklist.

Statistics

For all statistical analyses, confirm that the following items are present in the figure legend, table legend, main text, or Methods section.

n/a	Confirmed	
☐	☒	The exact sample size (*n*) for each experimental group/condition, given as a discrete number and unit of measurement
☐	☒	A statement on whether measurements were taken from distinct samples or whether the same sample was measured repeatedly
☐	☒	The statistical test(s) used AND whether they are one- or two-sided<br>*Only common tests should be described solely by name; describe more complex techniques in the Methods section.*
☒	☐	A description of all covariates tested
☒	☐	A description of any assumptions or corrections, such as tests of normality and adjustment for multiple comparisons
☐	☒	A full description of the statistical parameters including central tendency (e.g. means) or other basic estimates (e.g. regression coefficient) AND variation (e.g. standard deviation) or associated estimates of uncertainty (e.g. confidence intervals)
☐	☒	For null hypothesis testing, the test statistic (e.g. *F*, *t*, *r*) with confidence intervals, effect sizes, degrees of freedom and *P* value noted<br>*Give P values as exact values whenever suitable.*
☒	☐	For Bayesian analysis, information on the choice of priors and Markov chain Monte Carlo settings
☒	☐	For hierarchical and complex designs, identification of the appropriate level for tests and full reporting of outcomes
☐	☒	Estimates of effect sizes (e.g. Cohen's *d*, Pearson's *r*), indicating how they were calculated

Our web collection on statistics for biologists contains articles on many of the points above.

Software and code

Policy information about availability of computer code

Data collection	Flow cytometry: BD FACSDiva™ (9.0.1), Attune NxT Flow Cytometer (with autosampler)
Data analysis	FACS - flowjo (v10.8.2);Illumina amplicon sequencing: Crispresso2 (v2.0.20b) (Clement et al. 2019), Statistical analysis: GraphPad Prism 9.4.1, CRISPR screen analysis MaGECK (v 0.5.9.5) , drugZ (Hart-lab), Figure assembly- Adobe Illustrater, (v 28.5).

For manuscripts utilizing custom algorithms or software that are central to the research but not yet described in published literature, software must be made available to editors and reviewers. We strongly encourage code deposition in a community repository (e.g. GitHub). See the Nature Portfolio guidelines for submitting code & software for further information.

Data

Policy information about availability of data

All manuscripts must include a data availability statement. This statement should provide the following information, where applicable:

- Accession codes, unique identifiers, or web links for publicly available datasets
- A description of any restrictions on data availability
- For clinical datasets or third party data, please ensure that the statement adheres to our policy

Sequencing files for the pooled screen and endogenous genome editing in cell lines and primary cells are available in SRA BioProject PRJNA1117357. (https://www.ncbi.nlm.nih.gov/sra/PRJNA1117357)

Human research participants

Policy information about studies involving human research participants and Sex and Gender in Research.

Reporting on sex and gender	N/A
Population characteristics	N/A
Recruitment	N/A
Ethics oversight	N/A

Note that full information on the approval of the study protocol must also be provided in the manuscript.

Field-specific reporting

Please select the one below that is the best fit for your research. If you are not sure, read the appropriate sections before making your selection.

☒ Life sciences ☐ Behavioural & social sciences ☐ Ecological, evolutionary & environmental sciences

For a reference copy of the document with all sections, see nature.com/documents/nr-reporting-summary-flat.pdf

Life sciences study design

All studies must disclose on these points even when the disclosure is negative.

Sample size	No sample size calculations were performed. Sample size was determined to be adequate based on the consistency of measurable differences and previously published literature in the genome editing field. (see Anzalone, 2019, Nature, Moller, 2022, Nat Comm for example)
Data exclusions	Data were excluded from analysis in the case of failed experiments due to factors such as loss of reagent activity, contamination.
Replication	Each data point represents an individual biological replicate as listed in figure legends and all replications were successful.
Randomization	No randomization was performed. All independent biological experiments were treated equally.
Blinding	No blinding was performed since the data is not subjective.

Reporting for specific materials, systems and methods

We require information from authors about some types of materials, experimental systems and methods used in many studies. Here, indicate whether each material, system or method listed is relevant to your study. If you are not sure if a list item applies to your research, read the appropriate section before selecting a response.

Materials & experimental systems

n/a	Involved in the study	
☐	☒	Antibodies
☐	☒	Eukaryotic cell lines
☒	☐	Palaeontology and archaeology
☒	☐	Animals and other organisms
☒	☐	Clinical data
☒	☐	Dual use research of concern

Methods

n/a	Involved in the study	
☒	☐	ChIP-seq
☐	☒	Flow cytometry
☒	☐	MRI-based neuroimaging

Antibodies

Antibodies used	Primary antibodies: anti-TREX1 (abcam ab185228), -ß-actin (abcam ab8224), -RPA32 (abam ab2175) , -flag (abcam f1804) and -HSP60 (Santa Cruz Biotechnology sc-1052)<br>Secondary antibodies: Goat anti-Mouse IgG HRP (Thermo Fisher Scientific 31432) , Donkey anti-rabbit IgG HRP (SouthernBiotech 6441-05) , anti-rabbit secondary antibody IRDye 800 CW (LI-COR Biosciences (926-32213)) and anti-goat IRDye 800 CW (LI-COR Biosciences (926-32214))

Validation	No further validation is performed for the commercial antibodies which are highly used in different publications . Specifically TREX1 antibody is validated with KO clones in figure 2B. Anti-Flag antibody is validated in figure 5C.

Eukaryotic cell lines

Policy information about cell lines and Sex and Gender in Research

Cell line source(s)	Healthy donor, FANCA–/– (FA55) and FANCD2–/– (FA-75) lymphoblastic cell lines (LCLs) were generously gifted by Dr. Paolo Rio, CIEMAT. HEK293T, K562, Jurkat, U2OS, MD-MBA-431, RPE1, HELA were obtained from ATCC or Berkeley Cell Culture facility
Authentication	via STR
Mycoplasma contamination	All cell lines were routinely tested for mycoplasma (MycoAlert; Lonza) and tested negatively.
Commonly misidentified lines (See ICLAC register)	No commonly misidentified lines were used in this study.

Flow Cytometry

Plots

Confirm that:

☒ The axis labels state the marker and fluorochrome used (e.g. CD4-FITC).

☒ The axis scales are clearly visible. Include numbers along axes only for bottom left plot of group (a 'group' is an analysis of identical markers).

☒ All plots are contour plots with outliers or pseudocolor plots.

☒ A numerical value for number of cells or percentage (with statistics) is provided.

Methodology

Sample preparation	Cells were minimally processed (trypsinized and resuspended in standard media)
Instrument	Flow cytometry: BD FACSDiva™ Software, Attune NxT Flow Cytometer (with autosampler)
Software	FlowJo v10.8.2
Cell population abundance	Analytical flow had >10kcells. Pooled screen sort strategy is described in Supplementary Figure 2.
Gating strategy	Cells were gated on FSC/SSC to identify live cell populations. All subsequent analysis was performed on the live cell population. Then, cells were analyzed for BFP and GFP positive channel and gate is drawn for the GFP positive population as an example of Supplementary Figure 1 and 3

☒ Tick this box to confirm that a figure exemplifying the gating strategy is provided in the Supplementary Information.
